# Efficacy and safety of human umbilical cord-derived mesenchymal stem cells in the treatment of refractory immune thrombocytopenia: a prospective, single arm, phase I trial

**DOI:** 10.1038/s41392-024-01793-5

**Published:** 2024-04-23

**Authors:** Yunfei Chen, Yanmei Xu, Ying Chi, Ting Sun, Yuchen Gao, Xueqing Dou, Zhibo Han, Feng Xue, Huiyuan Li, Wei Liu, Xiaofan Liu, Huan Dong, Rongfeng Fu, Mankai Ju, Xinyue Dai, Wentian Wang, Yueshen Ma, Zhen Song, Jundong Gu, Wei Gong, Renchi Yang, Lei Zhang

**Affiliations:** 1 State Key Laboratory of Experimental Hematology, National Clinical Research Centre for Blood Diseases, Haihe Laboratory of Cell Ecosystem, Tianjin Key Laboratory of Gene Therapy for Blood Diseases, CAMS Key Laboratory of Gene Therapy for Blood Diseases, Institute of Hematology & Blood Diseases Hospital, Chinese Academy of Medical Sciences & Peking Union Medical College, Tianjin, 300020 China; 2 Tianjin Institutes of Health Science, Tianjin, 301600 China; 3 National Clinical Research Center for Hematologic Diseases, Jiangsu Institute of Hematology, The First Affiliated Hospital of Soochow University, Suzhou, 215006 China; 4 National Engineering Research Centre of Cell Products, Tianjin Key Laboratory of Engineering Technologies for Cell Pharmaceutical, AmCellGene Engineering Co., Ltd, Tianjin, 300457 China; 5 School of Population Medicine and Public Health, Chinese Academy of Medical Sciences and Peking Union Medical College, 100730 Beijing, China

**Keywords:** Immunological disorders, Immunological disorders, Mesenchymal stem cells

## Abstract

Patients with refractory immune thrombocytopenia (ITP) frequently encounter substantial bleeding risks and demonstrate limited responsiveness to existing therapies. Umbilical cord-derived mesenchymal stem cells (UC-MSCs) present a promising alternative, capitalizing on their low immunogenicity and potent immunomodulatory effects for treating diverse autoimmune disorders. This prospective phase I trial enrolled eighteen eligible patients to explore the safety and efficacy of UC-MSCs in treating refractory ITP. The research design included administering UC-MSCs at escalating doses of 0.5 × 10^6^ cells/kg, 1.0 × 10^6^ cells/kg, and 2.0 × 10^6^ cells/kg weekly for four consecutive weeks across three cohorts during the dose-escalation phase, followed by a dose of 2.0 × 10^6^ cells/kg weekly for the dose-expansion phase. Adverse events, platelet counts, and changes in peripheral blood immunity were monitored and recorded throughout the administration and follow-up period. Ultimately, 12 (with an addition of three patients in the 2.0 × 10^6^ cells/kg group due to dose-limiting toxicity) and six patients were enrolled in the dose-escalation and dose-expansion phase, respectively. Thirteen patients (13/18, 72.2%) experienced one or more treatment emergent adverse events. Serious adverse events occurred in four patients (4/18, 22.2%), including gastrointestinal hemorrhage (2/4), profuse menstruation (1/4), and acute myocardial infarction (1/4). The response rates were 41.7% in the dose-escalation phase (5/12, two received 1.0 × 10^6^ cells/kg per week, and three received 2.0 × 10^6^ cells/kg per week) and 50.0% (3/6) in the dose-expansion phase. The overall response rate was 44.4% (8/18) among all enrolled patients. To sum up, UC-MSCs are effective and well tolerated in treating refractory ITP (ClinicalTrials.gov ID: NCT04014166).

## Introduction

Immune thrombocytopenia (ITP) is an acquired autoimmune hemorrhagic disease characterized by isolated thrombocytopenia, resulting from an imbalance of immune tolerance that leads to accelerated platelet destruction and impaired platelet production.^1–3^ The most common clinical manifestations of ITP include the arrest in megakaryocyte maturation and reduction in platelet count. Treatments for adult patients with ITP include glucocorticoids, intravenous immunoglobulin, TPO receptor agonist (TPO-RA), rituximab, splenectomy, and immunosuppressants.^4,5^ Refractory ITP refers to patients who have failed multiple therapies (including TPO-RA and rituximab) and/or have not responded or relapsed after splenectomy.^6,7^ These patients often have aggravated bleeding symptoms, a severe decline in quality of life, and increased mortality.^6,7^ However, the regimens recommended by the current guidelines have limited effectiveness in alleviating the situation, and patients with refractory ITP are in urgent need of novel therapies to overcome this dilemma.^8,9^

Mesenchymal stem cells (MSCs), also known as mesenchymal stromal cells, are pluripotent progenitor cells with regenerative and immunomodulatory properties.^10^ Since Hillard Lazarus’s first report in 1995 on treating hematologic malignancies with autologous bone marrow-derived progenitor stromal cells, the application of MSCs has gradually increased.^11^ Many preclinical and clinical studies have confirmed that MSCs can influence both innate and adaptive immune responses through paracrine or cell-to-cell contact mechanisms.^12,13^ Current data show that 10 mesenchymal stem cell products have been approved for marketing worldwide and are mainly used for immunoregulation and injury repair in graft-versus-host disease (GVHD), Crohn’s disease, myocardial infarction, osteoarthritis and other diseases.^14,15^ We and other researchers have previously found that MSCs from ITP patients showed defective haematopoietic support function, impaired proliferative capacity, and reduced suppression of activated T cells. Meanwhile, we compared and analyzed the characteristics of MSCs from different tissue sources through single-cell transcriptomic and proteomic sequencing, and found that perinatal mesenchymal stem cells had stronger immunosuppressive ability than bone marrow-derived and adipose-derived MSCs.^16^ Therefore, correcting the impaired autologous MSCs by drugs or infusion of exogenous umbilical cord-derived MSCs (UC-MSCs) may partly reshape the immune microenvironment in patients with ITP.^17,18^

In the past decade, several researchers have attempted small-sample studies on the treatment of ITP using UC-MSCs and achieved the expected results. In 2012, we first reported that two patients with chronic ITP showed effective improvement in platelet count and bleeding symptoms after the administration of UC-MSCs (4.0 × 10^5^ cells/kg).^19^ Five years later, Wang reported that four chronic ITP patients who received UC-MSC infusion with a total cell count ranging between 5.0 × 10^7^ and 1.0 × 10^8^ achieved complete remission, and three relapsed within one year.^20^ Although the effectiveness of UC-MSCs in treating ITP has been recognized to some extent, there is no consensus on the dosage of infusion, and there is a lack of support from standardized clinical trial research evidence.^21^

Given the limited research results on the clinical efficacy, optimal treatment timing and regimen of MSCs for treating patients with refractory ITP, we conducted a prospective clinical study to evaluate the safety and efficacy of UC-MSCs for treating refractory ITP patients and explored the appropriate dose of UC-MSC therapy.

## Results

### Patient characteristics

**Fig. 1:**
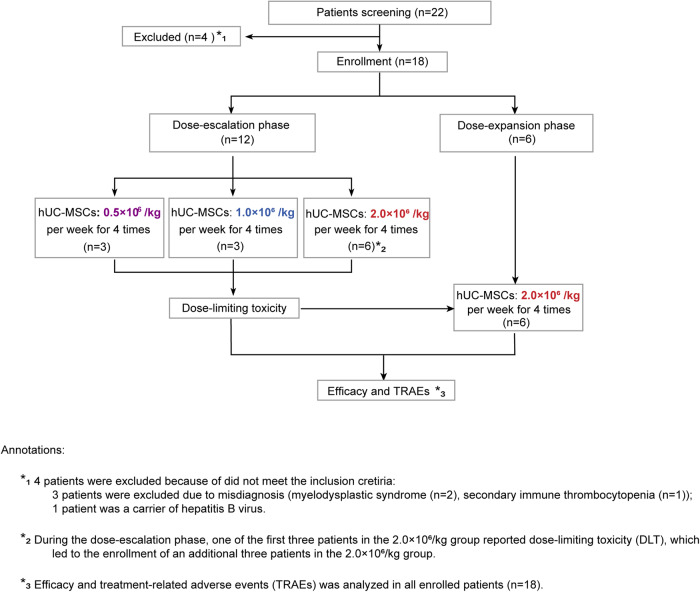
Flow diagram of the study. Twenty-two patients were screened for eligibility, 3 patients were excluded based on the eligibility criteria, and one patient withdrew informed consent. Finally, 18 patients were enrolled in the study. In the dose-escalation phase, 12 patients were enrolled and assigned to the 3-dose group, and 6 patients were subsequently enrolled in the dose-expansion phase

Between November 2019 and August 2022, 18 refractory ITP patients hospitalized in our center were successfully screened and enrolled in the study, including 12 patients in the dose escalation and 6 patients in the dose expansion phase (Fig. 1). The demographics and baseline characteristics of all eligible patients are shown in Table 1 and Supplementary Table S1. The median age of the patients was 44 years old (range: 30–50), and 15/18 patients (83.3%) were female. All of the participants were chronic ITP patients with a median duration of 73 months (range: 15–360). A total of 15/18 patients (83.3%) had 5 or more unique prior therapies (Supplementary Table S2). A total of 10/18 patients (55.6%) and 13/18 patients (72.2%) had previously received splenectomy and rituximab, respectively. Platelet glycoprotein (GP) autoantibodies were detected by a commercial kit (PakAutoassay, Immucor GTI Diagnostics, USA), and 11/18 patients (61.1%) showed positive results, including anti-GP Ib/IX single positive (2/18, 11.1%), anti-GP IIb/IIIa single positive (6/18, 33.3%), and anti-GP IIb/IIIa and GP Ia/IIa double positive (3/18, 16.7%).

**Table 1:** Demographic characteristics of eligible patients

Characteristics	Patients (*n* = 18)
Age (years), median (range)	44 (30–56)
Sex, *n* (%)	
Female	15 (83.3)
Male	3 (16.7)
Duration of thrombocytopenia (months), median (range)	73 (15–360)
Baseline platelet count (×10^9^/L), median (range)	6 (2–24)
<10 × 10^9^/L, *n* (%)	10 (55.6)
10–30 × 10^9^/L, *n* (%)	8 (44.4)
Previous therapies, *n* (%)	
Glucocorticoids	18 (100.0)
IVIG	17 (94.4)
rhTPO/TPO receptor agonists	17 (94.4)
Cyclosporine	7 (38.9)
Danazol	10 (55.6)
Rituximab	13 (72.2)
Splenectomy	10 (55.6)
Vincristine	5 (27.8)
Decitabine	3 (16.7)
Cyclophosphamide	1 (5.6)
Concomitant medications, *n* (%)	
Glucocorticoids	1 (5.6)
TPO receptor agonists	7 (38.9)
Danazol	1 (5.6)
Bleeding (WHO bleeding scale, grade 1–4), *n* (%)	18 (100.0)
Platelet glycoprotein (GP) autoantibodies, *n* (%)	
Anti-GP Ib/IX positive only	2 (11.1)
Anti-GP IIb/IIIa positive only	6 (33.3)
Anti-GP IIb/IIIa, GP Ia/IIa double positive	3 (16.7)
Negative	7 (38.9)

*IVIG* intravenous immunoglobulin, *rhTPO* recombination human thrombopoietin

### Safety and tolerability

During the dose-escalation phase, three patients were sequentially enrolled in each of the three predetermined dose groups and received UC-MSC treatment according to the protocol. Unfortunately, one patient in 2.0 × 10^6^ cells/kg group experienced a grade 3 treatment-related adverse event (acute myocardial infarction) 10 days after the fourth infusion of UC-MSCs, and it was determined by experts from the academic and ethics committee as a dose-limiting toxicity event (DLT) (Supplementary Fig. S1). Therefore, we added three more patients to the 2.0 × 10^6^ cells/kg group, and no DLT was reported in these patients. Based on this, we determined 2.0 × 10^6^ cells/kg as the dosage for the subsequent dose-expansion phase.

All 18 patients enrolled in the study successfully finished the full course (four times) of UC-MSC infusion. Within the 28-week UC-MSC treatment and follow-up period, 13 patients (13/18, 72.2%) had one or more treatment emergent adverse events (TEAEs), most of which were grade 1 or 2, and all of them recovered spontaneously or after short-term intervention. The detailed adverse events (AEs) are presented in Table 2. Briefly, the most common TEAEs were fatigue (5/18, 27.8%), blood bilirubin elevation (4/18, 22.2%) and uric acid elevation (3/18, 16.7%). Three patients reported treatment-related adverse events (TRAEs), including two cases of infusion-related reaction and one case of acute myocardial infarction that occurred ten days after the fourth infusion (with platelet count <30 × 10^9^/L at onset), which was considered possibly related to the infusion of UC-MSCs. Two patients (2/18, 11.1%) in 1.0 × 10^6^ cells/kg group experienced chest tightness that was spontaneously relieved during the first UC-MSC infusion.

**Table 2:** Treatment emergent adverse events (TEAEs) of UC-MSC infusion

TEAEs	Patients, *n* (%)				
	Grade 1	Grade 2	Grade 3	Grade 4–5	Any grade
Fatigue	5	–	–	–	5 (27.8)
Gastrointestinal hemorrhage		–	2	–	2 (11.1)
Profuse menstruation	1	–	1	–	2 (11.1)
Upper respiratory tract infection	2	–	–	–	2 (11.1)
Urinary tract infection	1	–	–	–	1 (5.6)
Furuncle	1	–	–	–	1 (5.6)
Myocardial infarction		–	1	–	1 (5.6)
Blood bilirubin elevation^a^	4	–	–	–	4 (22.2)
Alanine aminotransferase elevation	2	–	–	–	2 (11.1)
Aspartate aminotransferase elevation	1	–	–	–	1 (5.6)
Gamma-glutamyl transferase elevation	1	–	–	–	1 (5.6)
Alkaline phosphatase elevation	1	–	–	–	1 (5.6)
Blood uric acid elevation	3	–	–	–	3 (16.7)
Infusion related reaction	2	–	–	–	2 (11.1)

^a^Includes the total bilirubin, direct bilirubin, and indirect bilirubin elevation

Serious adverse events (SAEs) occurred in four patients (4/18, 22.2%), including patient 006 in 1.0 × 10^6^ cells/kg group, patients 007 and 009 in 2.0 × 10^6^ cells/kg group, and patient 014 in the expansion cohort. Patients 006 and 014 experienced grade 3 gastrointestinal hemorrhage, and patient 009 had profuse menstruation during UC-MSC treatment, these were all thought to be associated with thrombocytopenia. Patient 007 experienced the DLT event mentioned above. To date, all patients enrolled and treated with UC-MSCs have not reported any AEs of malignant neoplasms.

### Efficacy

The efficacy of UC-MSCs was analyzed separately for the dose-escalation phase and the dose-expansion phase (Supplementary Table S3). Excluding the influence of rescue therapy, the platelet response (R, defined as platelet count ≥30 × 10^9^/L, with at least 2-fold increase from the baseline count and the absence of bleeding) was achieved by 5 patients (41.7%, 5/12) in the dose-escalation phase, and 3 patients (50.0%, 3/6) in the dose-expansion phase. The overall response rate was 44.4% (8/18), with a median cumulative response duration of 6.5 weeks (range 3–27). The median time to the first platelet count ≥50 × 10^9^/L was 21 days (range: 7–42) (Supplementary Fig. S2).

Amone the 5 patients who achieved a response in the dose-escalation phase, 2 belonged to the 1.0 × 10^6^ cells/kg group, and 3 belonged to the 2.0 × 10^6^ cells/kg group. Therefore, the efficacy rate was 0.0% (0/3) for the 0.5 × 10^6^ cells/kg group, 66.7% (2/3) for the 1.0 × 10^6^ cells/kg group, and 50.0% (3/6) for the 2.0 × 10^6^ cells/kg group. The median cumulative response duration among responding patients was 4.5 weeks (range: 3–6) in the 1.0 × 10^6^ cells/kg group and 27 weeks (range: 7–27) in the 2.0 × 10^6^ cells/kg group, respectively. The proportions of patients with platelet counts ≥50 × 10^9^/L at least once were 0.0% (0/3) in the 0.5 × 10^6^ cells/kg group, 66.7% (2/3) in the 1.0 × 10^6^ cells/kg group, and 50.0% (3/6) in the 2.0 × 10^6^ cells/kg group. The proportions of patients with platelet counts ≥100 × 10^9^/L at least once were 0.0% (0/3), 66.7% (2/3), and 33.3% (2/6), respectively.

In the dose-expansion phase, three patients met a response, resulting in an efficacy rate of 50.0% (3/6). All three responding patients achieved platelet counts ≥50 × 10^9^/L at least once, with a median cumulative response duration of 6 weeks (range: 3–8). The proportions of patients with platelet counts ≥100 × 10^9^/L at least once was 50.0% (3/6).

**Fig. 2:**
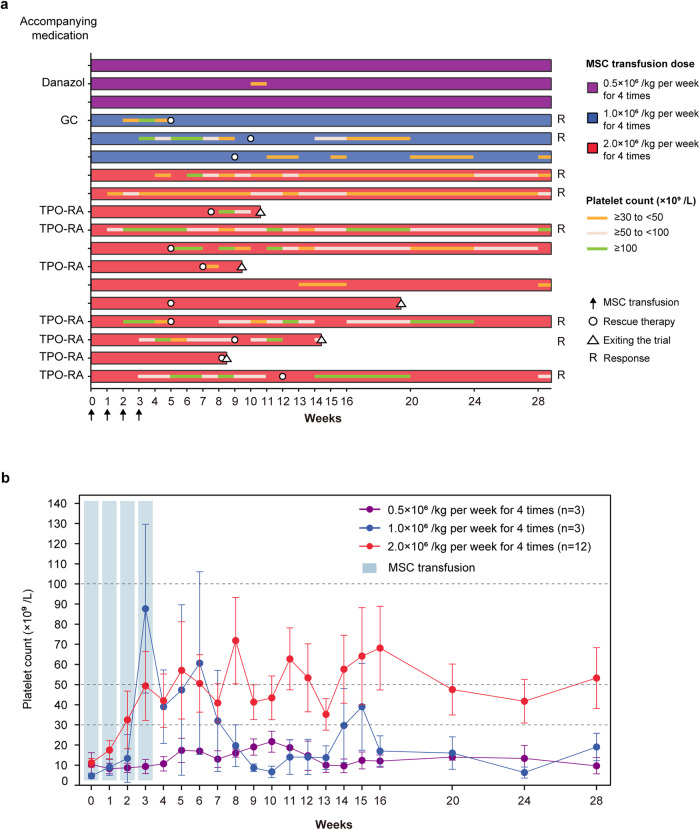
Treatment response, outcomes and platelet counts over time in all patients. **a** The best responses, concomitant medications, and rescue therapy of all 18 patients with different infusion doses (0.5 × 10^6^ cells/kg, 1.0 × 10^6^ cells/kg, and 2.0 × 10^6^ cells/kg) are shown in the swimmer plot. GC glucocorticoids, TPO-RA thrombopoietin receptor agonist, R response. **b** The mean platelet counts of all enrolled patients from baseline to 24 weeks after the completion of UC-MSC administration. Data are the mean ± s.e.m., and different colors represent different infusion doses of UC-MSCs

After four weeks of UC-MSC treatment, patients in each group showed an improvement in the bleeding symptoms (Supplementary Table S4). Six patients (6/12, 50.0%) and five patients (5/6, 83.3%) were treated with rescue therapy in the dose-escalation phase and dose-expansion phase, respectively (Supplementary Table S5). Nine patients (9/18, 50.0%) had concomitant medications in the study (Supplementary Table S6). Each patient’s concomitant medications, response and rescue therapy are shown as a bar chart in Fig. 2a. The mean platelet counts with standard errors from baseline to week 28 in all patients are shown in Fig. 2b.

Patient 008 relapsed 30 weeks after the first UC-MSC infusion, and patient 010 did not experience recurrence after one year of follow-up with eltrombopag 25 mg/day (the maintenance dose for three months before enrollment was 75 mg/day). Patient 007 relapsed 13 weeks after the first UC-MSC infusion, but the patient was no longer bleeding, and the platelet count was consistently >30 × 10^9^/L for 16 months of follow-up thereafter.

### Compassionate use of UC-MSCs

In total, we infused 19 courses of UC-MSCs in this study. Patient 008 received another course of UC-MSC (2.0 × 10^6^ cells/kg) treatment after relapse and reached treatment response again. However, the maximum platelet count after redosing was lower than that in the first course (61 × 10^9^/L vs. 89 × 10^9^/L), and the cumulative response time was also decreased (8 weeks vs. 30 weeks). TRAEs did not occur during the next 28 weeks.

### Pharmacokinetics of UC-MSCs

A total of eight patients underwent pharmacokinetics assays with informed consent (Supplementary Table S7). The SRY gene started to be detectable in the peripheral blood of all patients at 30 min and could last for 4 h in most patients. There was no detectable human-specific sequence in the blood 8 h after injection (except patient 005). Collectively, the longest peripheral blood residence time for MSCs administered via intravenous infusion in this study was 8 h.

### Humoral immunogenicity

Anti-drug antibodies (ADA) were analyzed pre-cell (−1 h ~ 0 h) and after-cell (48 h after the 4th MSC infusion) infusion in 12 patients (Supplementary Table S8). No ADA was detected at any time point, which demonstrated that there was no occurrence of ADA in patients after UC-MSC infusion.

### Immunological changes in the peripheral blood after UC-MSC treatment

**Fig. 3:**
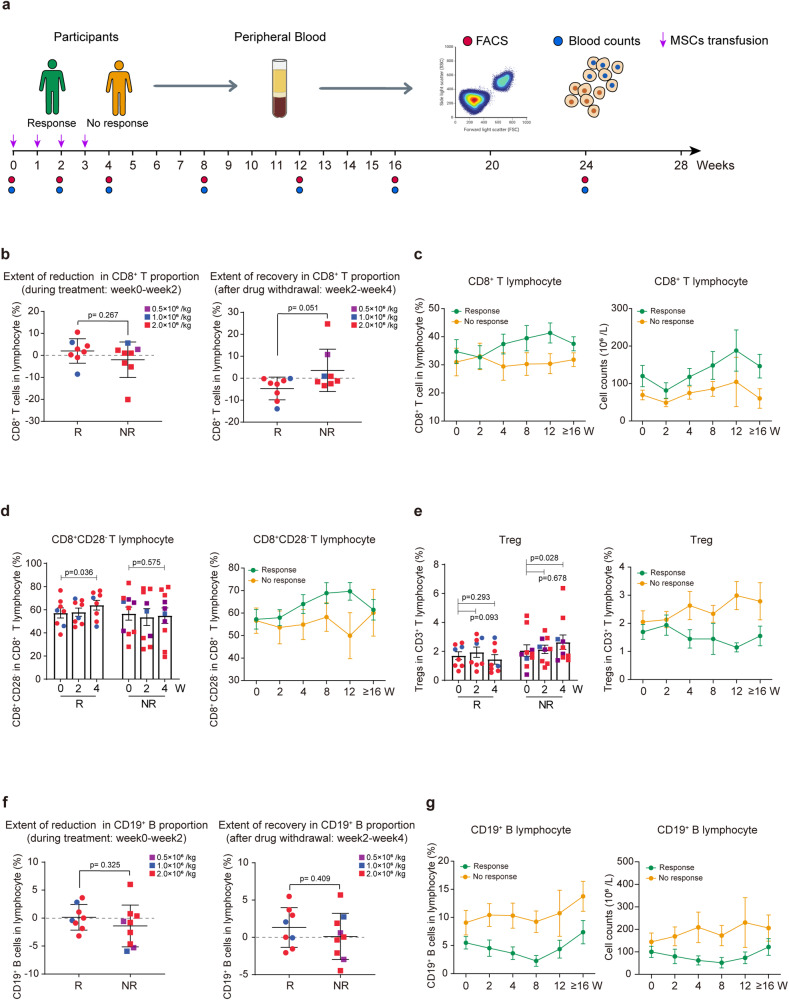
The peripheral immune monitoring plan of enrolled patients and the dynamic changes in peripheral blood immune cells after receiving UC-MSC infusion. **a** Blood samples were collected from all enrolled patients for platelet count and peripheral blood immune subpopulation monitoring at baseline and 2, 4, 8, 12, 16 and 24 weeks after the first infusion (peripheral blood samples at the baseline and 2 week observation points were collected within 2 h before the UC-MSC infusion). **b**, **c** Changes in the proportion of CD8+ T cells in peripheral lymphocytes after UC-MSC infusion (*n* = 8 in the response group, *n* = 8 in the no response group). **d**, **e** Changes in the proportion of suppressor T cells (Tregs, CD8^+^CD28^−^ T cells) after UC-MSC infusion (*n* = 8 in the response group, *n* = 10 in the no response group). **f**, **g** Dynamic changes in the counts and percentages of CD19^+^ B cells in peripheral blood after UC-MSC infusion (*n* = 8 in the response group, *n* = 9 in the no response group). Due to the impact of COVID-19, some follow-up data of enrolled patients were missing. Consequently, for the evaluation of peripheral immune alterations, we consolidated observations at 16 weeks and 24 weeks, presenting them as ≥16 weeks. Data are the mean ± s.e.m., and different colors represent different infusion doses of UC-MSCs in the scatterplots: purple = 0.5 × 10^6^ cells/kg, green = 1.0 × 10^6^ cells/kg, red = 2.0 × 10^6^ cells/kg. Statistical analysis was conducted using independent samples *t*-tests or Wilcoxon signed-rank test, with a significance level set at *p* < 0.05. W weeks, R response, NR no response

Differences in the peripheral immune environment of ITP patients before and after infusion were assessed through flow cytometry analysis (Fig. 3 and Supplementary Figs. S3–S8). Due to the impact of COVID-19, some follow-up data of the enrolled patients were missing. Consequently, for the evaluation of peripheral immune alterations, we consolidated observations at 16 weeks and 24 weeks, presenting them as ≥16 weeks.

Comparing baseline immune profiles between responsive and non-responsive patients, we found that responders displayed slightly higher percentages and absolute counts of T lymphocytes, whereas B cell metrics displayed the opposite trend. However, these variances lacked statistical significance (Supplementary Fig. S3). The infusion period of UC-MSCs was characterized by a transient reduction in both the proportion and absolute count of peripheral T lymphocytes. In the subsequent follow-up period, these metrics exhibited a gradual upward trend, especially in the proportion of CD8^+^ T cells, with this increase being slightly more evident in the response group compared to the no response group, though not reaching statistical significance (Fig. 3b, c and Supplementary Fig. S4a–g). Notably, a significant gradual increase in the proportion of CD8^+^CD28^−^ T cells was observed in the response group. Conversely, the proportion of T regulatory cells (Tregs, CD4^+^CD25^+^CD127^dim/−^) significantly rose in the no response group. The absolute counts of CD8^+^CD28^−^ T cells and Tregs demonstrated a similar pattern throughout the study period in both groups (Fig. 3d, e and Supplementary Figs. S5 and S6). Additionally, marginal decreases in the percentages of naïve (CD45RA^+^CCR7^+^ T cells) and central memory (CD45RA^−^CCR7^+^ T cells) T lymphocytes were observed in responsive patients (Supplementary Fig. S7). B cell monitoring revealed similar outcomes to T cells, though baseline B cell proportions were higher in non-responsive patients than in responsive patients (Fig. 3f, g and Supplementary Fig. S4h), with no significant difference in the suppressor B-cell subset (CD19^+^CD38^hi^CD24^hi^) between two groups (Supplementary Fig. S8).

**Fig. 4:**
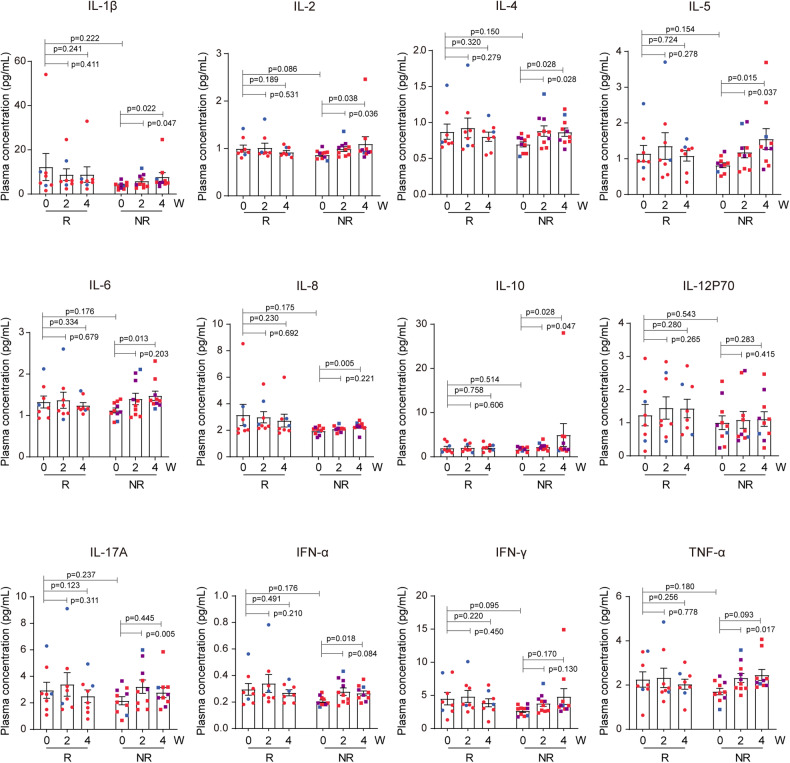
Quantitative analysis of plasma cytokine concentrations before and after UC-MSC infusion. The concentrations of 12 kinds of cytokines were measured before (0), during UC-MSC infusion (2 weeks), and after UC-MSC infusion (4 weeks) in the response and no response group, respectively (*n* = 8 in the response group, *n* = 10 in the no response group). The levels of plasma IL-1β, IL-2, IL-4, IL-5, IL-6, IL-8, IL-10, IL-17A, IFN-α, and TNF-α had significantly increased after the infusion of UC-MSC (all *p* < 0.05). No significant changes were observed in the response group before and after the UC-MSC infusion. Data are the mean ± s.e.m., circle represents response group, square represents no response group. And different colors represent different infusion doses of UC-MSCs in the scatterplots: purple = 0.5 × 10^6^ cells/kg, green = 1.0 × 10^6^ cells/kg, red = 2.0 × 10^6^ cells/kg. Statistical analysis was conducted using paired *t*-test or Wilcoxon signed-rank test, with a significance level set at *p* < 0.05. W weeks, R response, NR no response

Quantitative analysis of plasma cytokine concentrations before and after UC-MSC infusion (0, 2, 4 weeks) in patients revealed that there were no statistically significant differences in baseline cytokine levels between the response and no response groups. However, patients in the no response group showed a significant increase in plasma levels of various inflammatory cytokines, including IL-1β, IL-2, IL-6, and IL-17, after receiving UC-MSC infusion, whereas such changes were not observed in the response group (Fig. 4).

## Discussion

To our knowledge, this is the first prospective, open-label study that evaluated the safety and efficacy of UC-MSC infusion in refractory ITP and was first successfully documented by the China Medicinal Biotechnology Association. Although previous case reports and small sample studies have suggested that allogeneic MSC infusion may reshape immune homeostasis, improve platelet counts and alleviate bleeding symptoms in ITP patients, the sources of MSCs used in these studies vary greatly, and there are significant differences in the infusion doses used in different studies.^18,21^ In the dose-escalation phase of this study, we compared and analyzed the safety and efficacy of UC-MSC therapy for refractory ITP under three different doses and established 2.0 × 10^6^ cells/kg as the infusion dose for the dose-expansion phase.

As the most important study endpoint of this research, 72.2% of the enrolled patients experienced TEAEs after treatment, most of which were grade 1–2. Grade 3 bleeding manifestations (gastrointestinal hemorrhage, profuse menstruation) occurred in three patients, which may be attributable to the fact that all subjects in this study were refractory ITP patients with bleeding tendency and the limited efficacy of UC-MSCs. The DLT in this study was acute myocardial infarction, which was reported in one patient in the 2.0 × 10^6^ cells/kg group. Although there is no previous report that MSC infusion can induce thrombotic events, the platelet count on set was low, and there was no previous history of cardiovascular disease or risk factors, so UC-MSC infusion could not be ruled out.^22^ Notably, 22.2% of the subjects reported elevated liver transaminase (all incidents were Grade 1 AEs) during the observation period, which were considered related to the long-term use of concomitant drugs (danazol or TPO-RAs).

All 18 patients included in the study completed the UC-MSC treatment successfully, with an overall effective rate of 44.4% (8/18), and four patients (4/18, 22.2%) reached complete response after treatment. However, the 2.0 × 10^6^ cells/kg dosage did not show the expected advantage compared to the 1.0 × 10^6^ cells/kg dosage in achieving long-term remission (≥6 months, data not shown) after the infusion of UC-MSCs. Results in the dose-escalation phase reveal a dose-dependent trend in the efficacy of UC-MSCs in the dose range of 0.5–2.0 × 10^6^ cells/kg, but this tendency is less pronounced when analyzing data from both phases together. UC-MSC therapy is also effective in patients who have failed or relapsed after undergoing splenectomy or rituximab. The response rates among patients who had previously undergone splenectomy or rituximab were 40.0% (4/10) and 46.2% (6/13), respectively. Patients who had received both splenectomy and rituximab previously showed a similar response rate (40.0%, 2/5). In addition, our results indicated that bleeding symptoms can be completely relieved in 60.0%–75.0% of patients after treatment, supporting the feasibility of mesenchymal stem cells as an auxiliary or emergency treatment option.

In this study, we provided some insights into the circulation dynamics of UC-MSCs by detecting the SRY gene in peripheral blood. This approach minimizes patient discomfort and is ethically acceptable. However, it cannot provide information about the distribution and metabolism of UC-MSCs in organ tissues, which is a limitation of our research. We anticipate the development of more advanced methods in the future that can simultaneously monitor the distribution and metabolism of UC-MSCs in both peripheral blood and tissues. Such advancements will contribute to a more comprehensive understanding of the potential mechanisms underlying UC-MSC treatment for refractory ITP and variations in treatment efficacy among individuals.

Previous in vitro and in vivo studies have shown that mesenchymal stem cells exert immunomodulatory effects mainly through paracrine or direct cell contact, and the main target cells are T cells.^23,24^ In this study, we monitored peripheral blood immune cell subsets before and after UC-MSC infusion. Our findings indicate a temporary decrease in T cell percentages during the infusion of UC-MSCs, followed by a gradual increase in the proportion of CD8^+^CD28^−^ suppressive T cell subsets. These observations would lend further support to the notion that the immunosuppressive actions of MSCs in vivo are predominantly directed at T cells, particularly in attenuating the activation of CD8^+^ T cells. Nonetheless, given the limited sample size and variability in dosage among patients, these results warrant confirmation through larger-scale, randomized controlled trials. In addition, we also observed a decrease in the proportion of B cells after infusion, which was considered to be associated with the inhibition of T cells by UC-MSCs indirectly affecting the activation of B cells.^25^ The immunomodulatory capacity of MSCs is plastic and is believed to depend upon the kinds and concentration of inflammatory medicators as well as the intensity of the immune microenvironment activation.^26^ Quantitative analysis of peripheral blood plasma cytokines revealed a predominant elevation in proinflammation cytokines in the no response group, which implied that the failure of UC-MSC treatment might be partly associated with the intricate state of the inflammation microenvironment.

This study has several limitations. Firstly, it is a single-arm, non-randomized controlled trial with a small sample size. Additionally, the total duration of follow-up was relatively short, and the COVID-19 pandemic resulted in missing data at certain follow-up points for some participants. Moreover, the limited sample size precluded the possibility of conducting subgroup analyses, including those pertinent to changes in peripheral blood immune status post-UC-MSC infusion. Therefore, the results pertaining to safety and efficacy reported in this study should be validated through future large-scale, randomized, multicentre clinical trials.

Overall, UC-MSC infusion achieved a 44.4% overall response rate in patients with refractory ITP and mild adverse events. The related mechanism may involve inhibiting T-cell activation and inducing the production of suppressor CD8+ T cells. However, given the limited sample size and expansion dosage of this study, the results need to be further verified by clinical trials with larger sample sizes and higher infusion doses.

## Methods

### Study design

This prospective, open-label, phase I study consisted of two parts: a dose-escalation phase for exploring the safety and efficacy of three different dosage groups according to the traditional 3 + 3 protocol and a dose-expansion phase for further verifying the optimal UC-MSC dosage (Fig. 1).^27,28^ The study protocol was approved by the Ethics Committees of the Institute of Hematology and Blood Diseases Hospital, and informed consent was obtained from each participant according to the Declaration of Helsinki (ClinicalTrials.gov ID: NCT04014166).

### UC-MSC product preparation

Off-the-shelf GMP-grade male infant-derived UC-MSCs were provided by Tianjin Amcellgene Co. Ltd., which has been approved by the China National Medical Products Administration (NMPA) with agreements to start clinical trials for GVHD and acute chronic liver failure (ACLF). This cryopreserved product is a mixture containing 2.0 × 10^7^ UC-MSCs, 10% DMSO, and 5% human serum albumin, which needs to be thawed in a 37 °C water bath and diluted with multiple electrolyte injections (20 ml) before infusion into patients.

### Patients and treatments

Patients diagnosed with refractory ITP according to the international consensus on the identifying and treating of refractory immune thrombocytopenia were screened for the study.^7,29^ The major inclusion criteria included age 18 to 60 years, ITP duration lasting for more than 6 months, platelet count <30 × 10^9^/L and concomitant bleeding manifestations at enrollment. Patients were allowed to have concomitant treatments (TPO-RAs, glucocorticoids, danazol), but the doses must have been stable at the time of enrollment. Patients who had thrombosis, malignant tumors or severe dysfunction of vital organs (liver, kidney, lung, heart) were excluded from the study (see Supplementary Methods for details).

In the dose escalation phase, eligible patients were divided into three different dose groups sequentially. The UC-MSCs were administered once a week for a total of four times (0.5 × 10^6^ cells/kg group; 1.0 × 10^6^ cells/kg group; 2.0 × 10^6^ cells/kg group). Subsequently, the investigator determined an optimal dose to expand the sample size by referring to the results of the dose escalation phase. In addition, during the UC-MSC infusion and follow-up period, the dosage of concomitant medications should be kept stable or gradually tapered. If the participants had sustained thrombocytopenia (platelet count <30 × 10^9^/L) or severe bleeding manifestations during the follow-up period, the investigators should propose appropriate rescue therapy or advise the patient to withdraw from the study. Moreover, if the patient did not respond well to all of the current ITP regimens and was willing to have UC-MSCs administered again, a course of UC-MSC treatment could be given again 16 weeks after the first infusion.

Patients needed to visit every week from day 1 to day 112 and every month for three months thereafter to collect data on safety and efficacy outcomes, pharmacokinetic data of UC-MSCs, incidence of UC-MSC antibodies and changes in immune status.

### Pharmacokinetic study

An exploratory pharmacokinetic study was conducted to clarify the retention time of UC-MSCs in peripheral blood after injection. Briefly, the blood samples of female subjects who had never gave birth to a male infant were collected before (−1 h～0 h) and after (30 min, 1, 2, 4, 8, 16, 24, 48, 72, and 96 h) UC-MSCs infusion. Then, total DNA was extracted for PCR assays to detect the specific human DNA sequence as the SRY gene according to the manufacturer’s instructions. The operation range of the standard curve should have covered 4.00 × 10^1^–1.00 × 10^7^ copies/reaction.

### Anti-MSC antibody detection assay

The GMP-grade UC-MSCs were dissociated into single cells by 0.25% Trypsin-EDTA (Gibco), incubated with ADA samples for up to 30 min, and then labeled with conjugated antibodies against protein L-PE (Sino Biological) for 15 min in the dark. After washing with PBS twice, the cells were analyzed by FACS Canto II (BD Biosciences, USA) according to the instructions.

### Cell staining and flow cytometry

To monitor the influence of UC-MSC infusion on peripheral immune cells, blood samples were collected from patients at various monitoring points (Fig. 3a) and stained with antibodies (Supplementary Methods). Then, the samples were lysed with lysis buffer (BD Biosciences, USA) and washed twice with PBS. Stained cells were analyzed by FACS Canto II cytometry (BD Biosciences, USA). Quantitatively analyzed the concentrations of 12 cytokines (IL-1β, IL-2, IL-4, IL-5, IL-6, IL-8, IL-10, IL-12, IL-17, IFN-γ, IFN-α, and TNF-α) in peripheral blood plasma using flow cytometry bead-based assay (ABplex Human Chemokine 12-Plex Assay Kit, ABclonal).

### Outcomes

The primary endpoints were the safety and tolerability of UC-MSCs, which were reflected by the DLT. The National Cancer Institute Common Terminology Criteria for Adverse Events version 5.0 was used as the principle for grading and recording AEs throughout the study. The main evaluation indexes consist of physical examination, laboratory tests (liver function, renal function, electrocardiography, and virological examination) and infusion-related reactions. DLT was defined as any grade ≥3 TRAEs that occurred during the first administration to 1 month post completion of UC-MSCs and could not be relieved to grade 1 within 72 h after treatment with drugs or other interventions. The correlation between treatment and AEs was determined by the principal investigator. The secondary endpoints were the efficacy, pharmacokinetic and immunological data of UC-MSCs. Clinical response was evaluated according to the efficacy criteria of ITP, as outlined in the Supplementary Methods.^30^ Response (R) was defined as platelet count ≥30 × 10^9^/L, with at least 2-fold increase from the baseline count and the absence of bleeding. No response (NR) was defined as platelet count <30 × 10^9^/L or less than 2-fold increase from the baseline platelet count or the presence of bleeding. Relapse was defined as a platelet count <30 × 10^9^/L, or less than a doubling of the baseline count, or bleeding, which occurring after the achievement of a response. The efficacy endpoint was the proportion of patients with platelet count ≥30 × 10^9^/L at least doubling of the baseline count, ≥50 × 10^9^/L, and ≥100 × 10^9^/L at least once after receiving UC-MSCs. Changes in WHO bleeding score after treatment were assessed based on the World Health Organization’s Bleeding Scale.^31^

### Statistical analysis

All statistical analyses were mainly descriptive and conducted using SPSS v26.0 and GraphPad Prism 9.0 software. Quantitative data are presented as medians (minimum and maximum), and qualitative data are presented as *n* (%). Statistical differences in the immunological changes between the effective and ineffective groups were calculated using independent sample *t*-test, paired *t*-test or Wilcoxon signed-rank test, with *p* values less than 0.05 considered statistically significant. Summary tables for AEs had to include all AEs that occurred in the whole period of study.

### Supplementary information

Supplementary Information

protocol

## Data Availability

Eligible researchers can request data sets, including unidentified individual subject data. Data may be available on request from the corresponding author from 12 to 36 months after trial completion.
