# Supplementary material for: Efficacy and safety of human umbilical cord-derived mesenchymal stem cells in the treatment of refractory immune thrombocytopenia: a prospective, single arm, phase I trial
Source: Signal Transduct Target Ther. 2024 Apr 23;9:102. doi: 10.1038/s41392-024-01793-5 (PMC11039759; doi:10.1038/s41392-024-01793-5)
Supplement: Supplementary file 1 — Supplementary Information [file 41392_2024_1793_MOESM1_ESM.docx]

Supplementary Materials for

**Efficacy and Safety of Human Umbilical Cord-Derived Mesenchymal Stem Cells in the Treatment of Refractory Immune Thrombocytopenia: A Prospective, Single Arm, Phase I Trial**

Yunfei Chen, Yanmei Xu, Ying Chi, Ting Sun, Yuchen Gao, Xueqing Dou, Zhibo Han, Feng Xue, Huiyuan Li, Wei Liu, Xiaofan Liu, Huan Dong, Rongfeng Fu, Mankai Ju, Xinyue Dai, Wentian Wang, Yueshen Ma, Zhen Song, Jundong Gu, Wei Gong, Renchi Yang, Lei Zhang

Correspondence to: zhanglei1@ihcams.ac.cn

**This PDF file includes:**

Supplementary Methods

Figures. S1 to S8

Tables S1 to S8

1. **Supplemental methods**
   1. **Inclusion and exclusion criteria**
      1. **Inclusion criteria****:**

(1) 18-60 years of age;

(2) Confirmed diagnosis of ITP for at least 6 months with an insufficient response to first-line treatment drugs (IVIG, glucocorticoids), and thrombopoietic agents (recombinant human thrombopoietin (rhTPO), eltrombopag, avatrombopag, romiplostim, etc.) and anti-CD20 monoclonal antibody (rituximab) in second-line, or failed to splenectomy or subsequent relapse;

(3) Subjects who received splenectomy or rituximab must have finished the therapy more than 3 months prior to UC-MSCs inclusion;

(4) Platelet counts < 30×10^9^/L with bleeding;

(5) Expected survival time for at least 6 months;

(6) The function of liver and kidney is less than 1.5 times the upper limit of normal value, and the physical examination is qualified;

(7) Subjects are allowed to maintain stable ITP concomitant treatment. The dose of glucocorticoids (less than or equal to 0.5mg/kg prednisone or equivalent dose of glucocorticoids) and thrombopoietin receptor agonists should be stable for at least 4 weeks, and the dose of azathioprine, danazol, cyclosporine A, mycophenolate mofetil, tacrolimus, and retinoic acid should be stable for at least 12 weeks;

(8) Be able to understand the purpose and risks of the study and provide informed consent in accordance with national and local privacy regulations;

(9) Cardiac function: Grade 2 or below according to the New York Society for Cardiac function;

(10) ECOG physical status score ≤ 2;

- - 1. **Exclusion criteria:**

(1) Subjects with primary disease of important organs (liver, kidney, heart, etc.), or with immune system diseases;

(2) Secondary thrombocytopenia caused by various reasons, such as connective tissue disorders, bone marrow hematopoietic failure disease, myelodysplastic syndrome, malignancy, drugs, etc.;

(3) Subjects infected with human immunodeficiency virus (HIV);

(4) Uncontrollable and active infections during the screening period, including hepatitis B, hepatitis C, cytomegalovirus, EB virus, or positive syphilis antigen;

(5) Subjects with extensive and severe bleeding, such as hemoptysis, upper gastrointestinal hemorrhage, intracranial hemorrhage;

(6) Subjects with heart disease that requires treatment or hypertension that has been judged by researchers to be poorly controlled currently;

(7) Subjects with any venous or arterial thrombosis, atherosclerosis, and other diseases;

(8) Subjects with a history of malignant solid tumor or have received allogeneic stem cell transplantation or organ transplantation;

(9) Subjects with mental disorders who are unable to sign normal informed consent and conduct trials and follow-up;

(10) Subjects whose toxic symptoms caused by pre-trial treatment have not disappeared;

(11) Subjects with other serious diseases that may limit their participation in this trial (diabetes; severe cardiac insufficiency; myocardial obstruction or unstable arrhythmia or unstable angina pectoris in the last 6 months; gastric ulcer; active autoimmune disease, etc.);

(12) Subjects with septicemia or other irregular bleeding;

(13) Female subjects who are nursing or pregnant/suspected pregnant (positive pregnancy tests for human chorionic gonadotropin in urine during screening).

- 1. **Definition of response**

1. Response (R) was defined as platelet count ≥30×10^9^/L, with at least 2-fold increase from the baseline count and the absence of bleeding.
2. Complete response (CR) was defined as platelet count ≥100×10^9^/L and the absence of bleeding.
3. No response (NR) was defined as platelet count <30×10^9^/L or less than 2-fold increase from the baseline platelet count or the presence of bleeding.
4. Relapse was defined as a platelet count < 30 × 10^9^/L, or less than a doubling of the baseline count, or bleeding, which occurring after the achievement of a response.
5. When defining a CR or R, it should be tested at least twice with an interval of at least 7 days, and 1 day apart to define NR or relapse.
   1. **Antibodies**

| **Format** | **Molecule name** | **Source** |
| --- | --- | --- |
| FITC | CD19, CD3, CD4, CD45RO, CD14 | Biolegend |
| APC | CD19, CD3, CD24, CD127, CD28, CD80 | Biolegend |
| PE | CD8, CD27, CD38, CD25, CD86 | Biolegend |
| PerCP | CD45RA, CD40 | Biolegend |
| PE/Cy7 | CCR7, CD69 | Biolegend |

1. **Supplemental figures**

**Fig. S1: Dynamic changes in platelet counts over time in patient 007 after receiving UC-MSC infusion.**

**
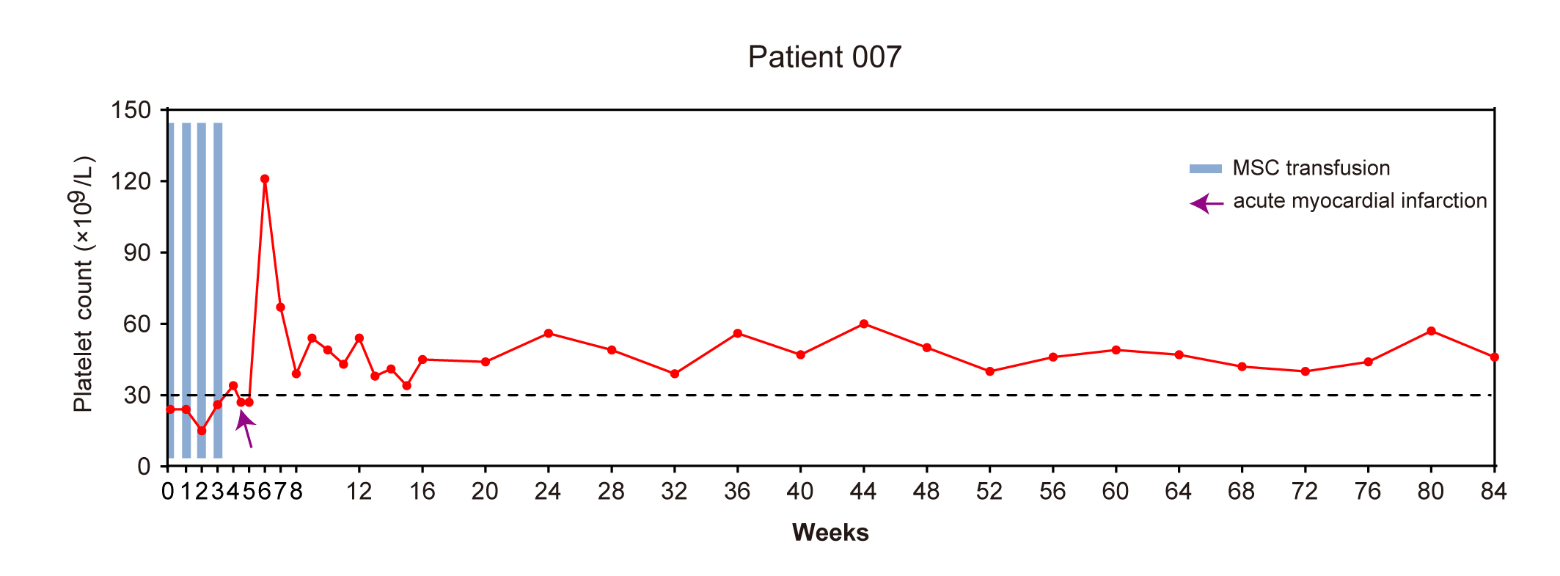
**

Patient 007 successfully completed the infusion of UC-MSCs, the platelet counts did not show apparently increase in the first four weeks. In the middle of the fourth week (indicated by the arrow), the patient had sudden chest tightness and precordial discomfort, and the electrocardiogram showed inferior subepicardial myocardial injury; serum troponin T 1.65 ng/ml (normal range 0.02-0.13 ng/ml); platelet count 27×10^9^/L; coagulation function was normal. Considering that the patient had acute myocardial infarction, doctors prescribed isosorbide dinitrate, the patient’s pain gradually relieved, and no other adverse events were reported during subsequent visits.

**Fig. S2: Cumulative incidence plots of patients who responded to the UC-MSC treatment.**

**
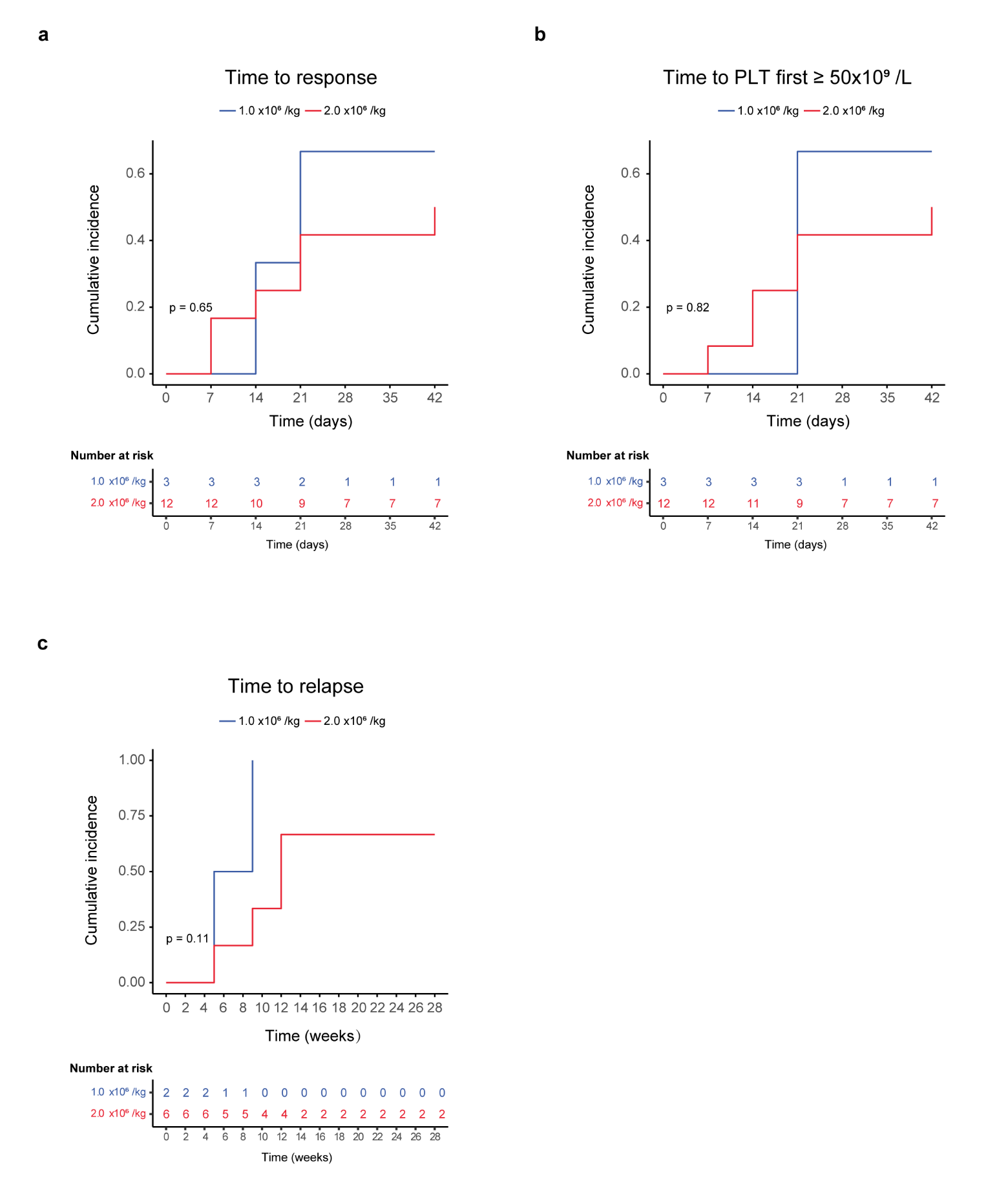
**

**(a)** The time to platelet count ≥30×10^9^/L and twice the baseline was similar between the two group. **(b)** The time to first platelet count ≥50×10^9^/L was similar between the two group. **(c)** Responders in the 2.0×10^6^ cells/kg UC-MSC infusion (in red) showed a similar trend of longer cumulative duration of a platelet count of ≥30×10^9^/L and twice the baseline compared to the 1.0×10^6^ cells/kg group (in blue). These plots were mainly generated using the R package “survmine”.

**Fig. S3: Baseline lymphocyte proportions and absolute counts among response and no response group.**

**
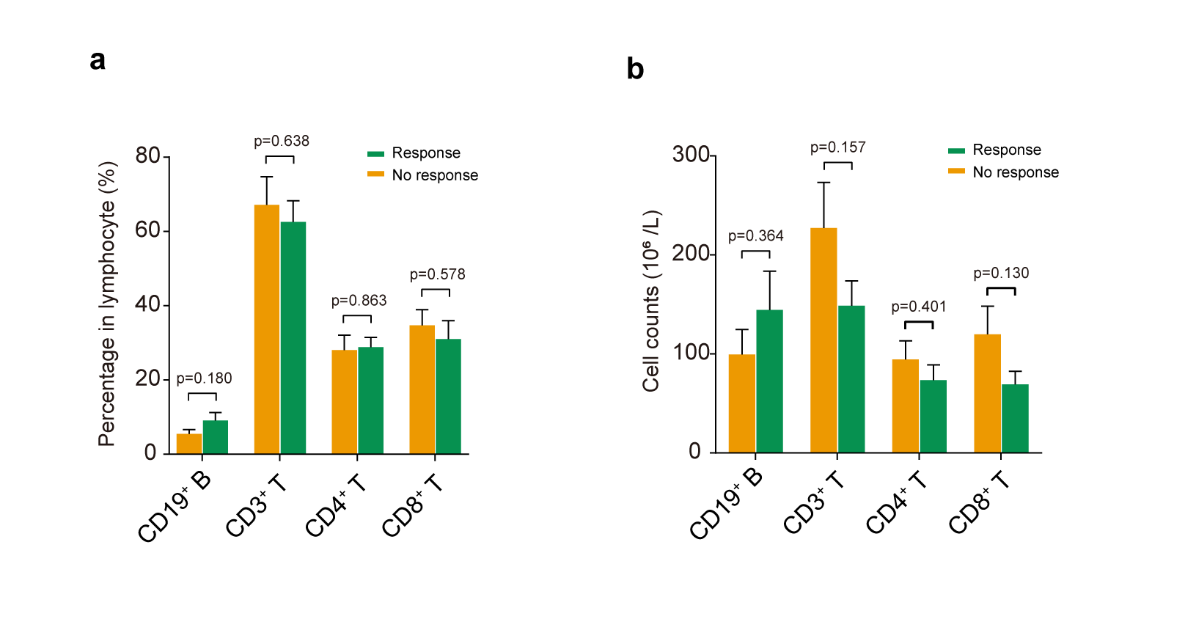
**

**(a)** Baseline counts of CD19^+^ B lymphocytes in response patients were lower than in no response patients, baseline counts of CD3^+^ T, CD4^+^ T and CD8^+^ T lymphocytes in response patients were higher than in no response patients. **(b)** Baseline percentage of lymphocytes showed similar tendency to counts of lymphocytes in response and no response patients. n=8 in response group, n=9 in no response group. Data are mean ± s.e.m.. Statistical analysis was conducted using independent samples t-tests, with a significance level set at *p* <0.05.

**Fig. S4: Dynamic alterations in T lymphocyte percentages and cell counts of lymphocytes among response and no response group during the whole study period.**


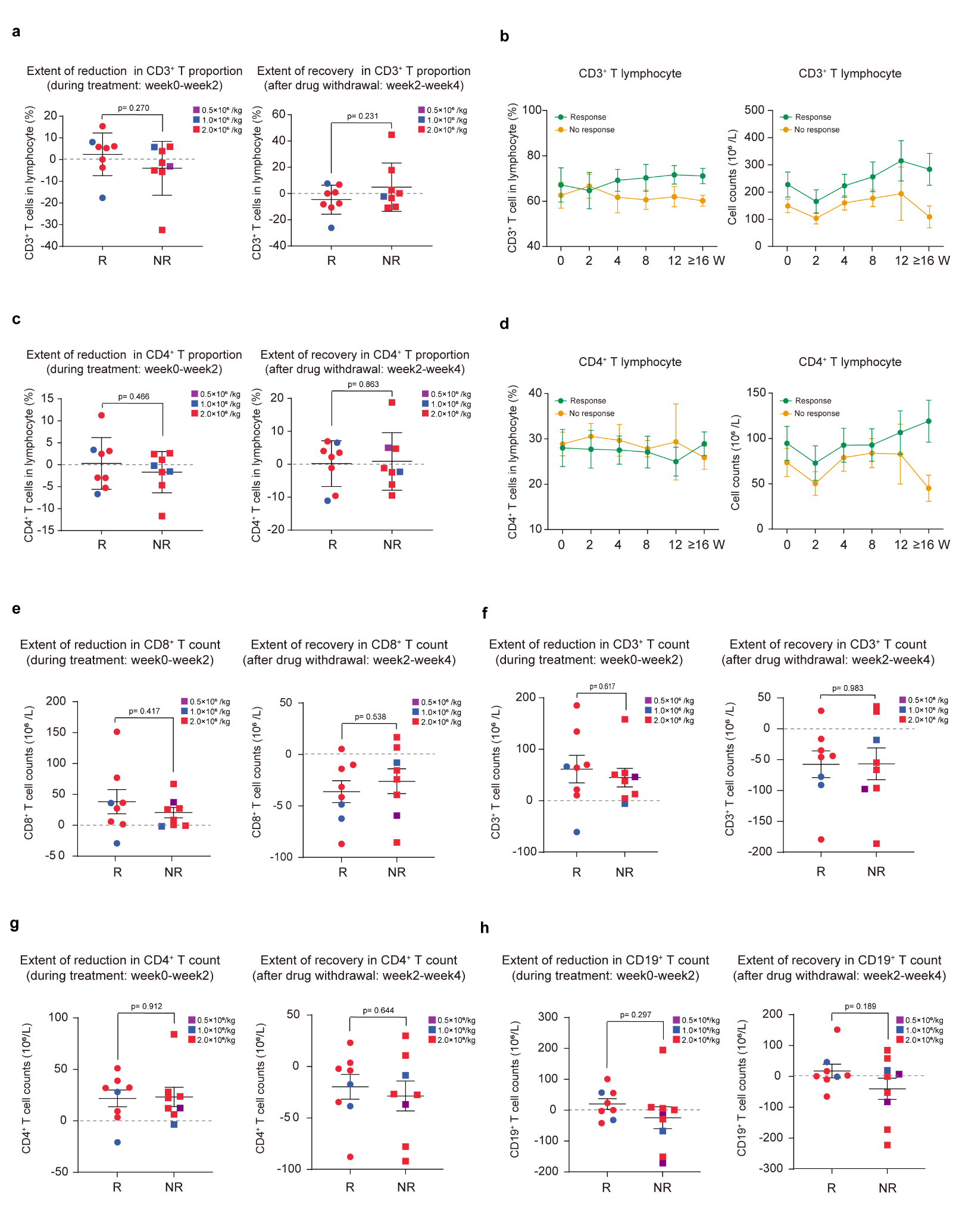


**(a-d)** Changes in the proportions and absolute counts of CD3^+^ T and CD4^+^ T lymphocytes following the UC-MSC treatment (n=8 in response group, n=8 in no response group). **(c-g)** Changes in the absolute counts of CD3^+^ T, CD4^+^ T, and CD8^+^ T lymphocytes during the UC-MSC treatment period (n=8 in response group, n=8 in no response group). **(h)** Dynamic changes in the absolute counts of CD19^+^ B lymphocytes during the UC-MSC treatment period (n=8 in response group, n=9 in no response group). Data are mean ± s.e.m., and different colours represent different infusion doses of UC-MSCs in the scatterplots: purple=0.5×10^6^ cells/kg, green=1.0×10^6^ cells/kg, red=2.0×10^6^ cells/kg. Statistical analysis was conducted using independent samples t-tests, with a significance level set at *p* <0.05. W: weeks, R: response, NR: no response.

**Fig. S5: Dynamic alterations in cell counts of suppressor CD8^+^ T cells and Treg following UC-MSC infusion.**

**
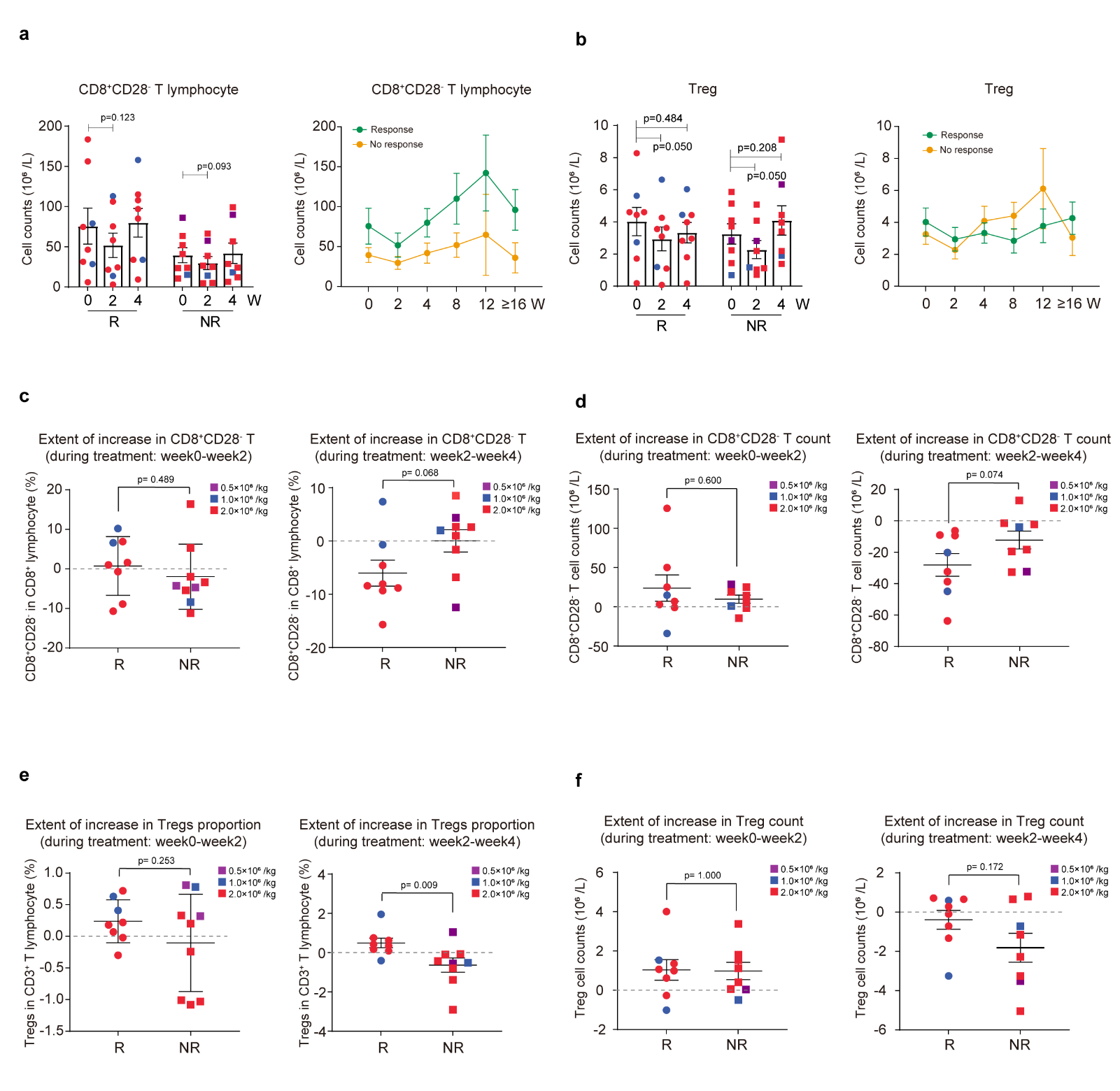
**

**(a)** The absolute counts of CD8^+^CD28^-^ T cells in patients undergoing UC-MSC infusion showed a temporary decline, followed by a steady increase following the commencement of UC-MSC treatment. **(b)** The absolute counts of Treg displayed a temporary decline during the UC-MSC treatment period. **(c-d)** Changes of CD8^+^CD28^-^ T cell proportions and absolute counts during the UC-MSC infusion period among the response and no response group. **(e-f)** Changes of Treg proportions and absolute counts during the UC-MSC infusion period among the response and no response group. Different colours represent different infusion doses of UC-MSCs in the scatterplots: purple=0.5×10^6^ cells/kg, green=1.0×10^6^ cells/kg, red=2.0×10^6^ cells/kg. Statistical analysis was conducted using independent samples t-tests, with a significance level set at *p* <0.05. W: weeks, R: response, NR: no response.

**Fig. S6: Percentage of Treg and suppressor CD8^+^ T cells increased after UC-MSC infusion.**

**
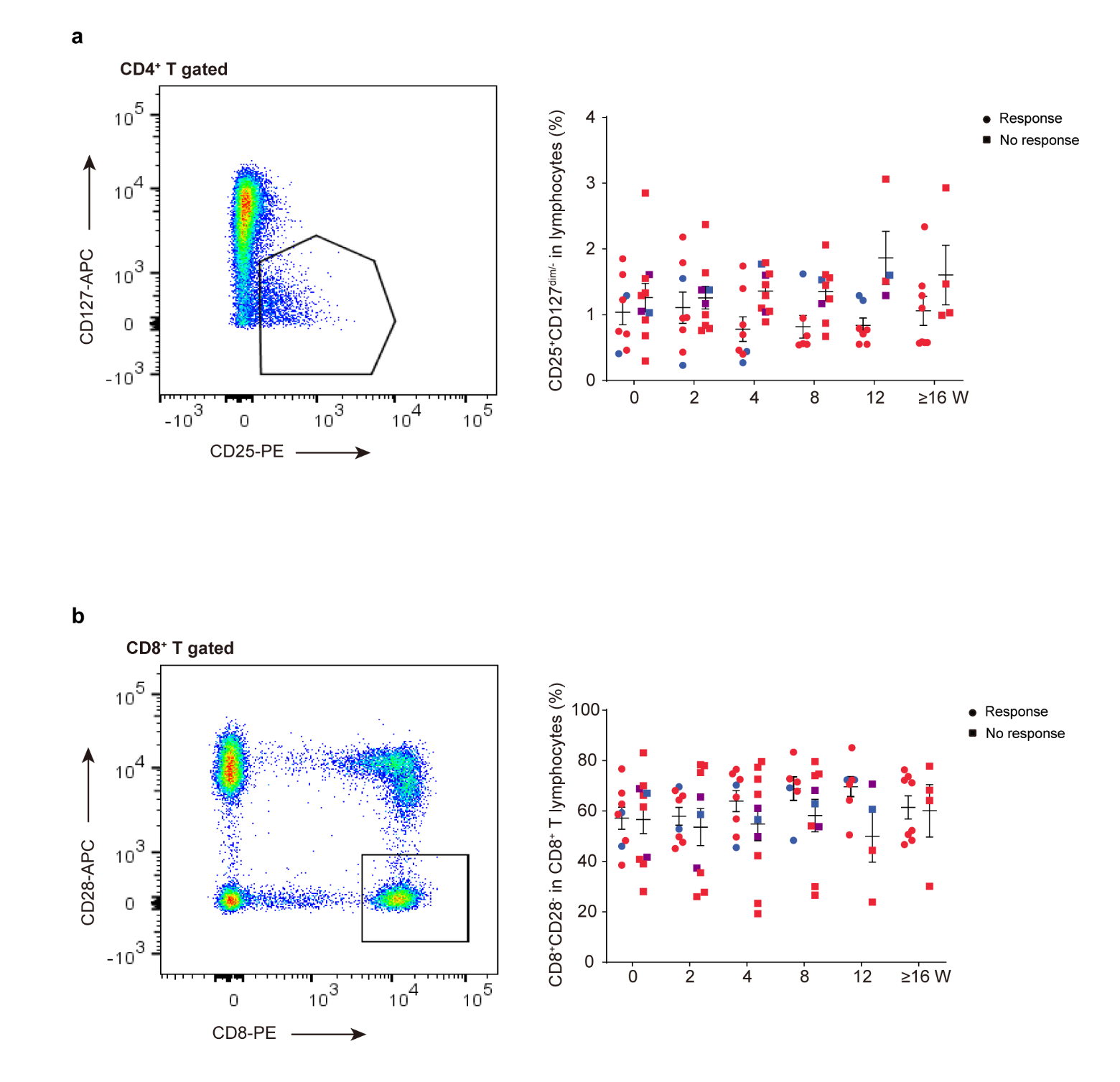
**

**(a)** Treg was gated by CD4^+^CD25^+^CD127^dim/-^ (left), and the percentage of Treg in lymphocytes transiently increased during UC-MSC infusion, and quickly decreased after the complement of treatment in both two groups (right). **(b)** Gated CD8^+^CD28^-^ T cells as suppressor CD8^+^ T cells (left). The percentage of CD8^+^CD28^-^ T cells in CD8^+^ T cells increased slowly and continuously in both response and no response group after UC-MSCs infusion, which was more obvious in the response group (right). Data are mean ± s.e.m (n=8 in response group, n=10 in no response group). Different colours represent different infusion doses of UC-MSCs in the scatterplots: purple=0.5×10^6^ cells/kg, green=1.0×10^6^ cells/kg, red=2.0×10^6^ cells/kg. W: weeks.

**Fig. S7: Dynamic changes in the proportion of T cell subset in the response and no response group after UC-MSC infusion.**

**
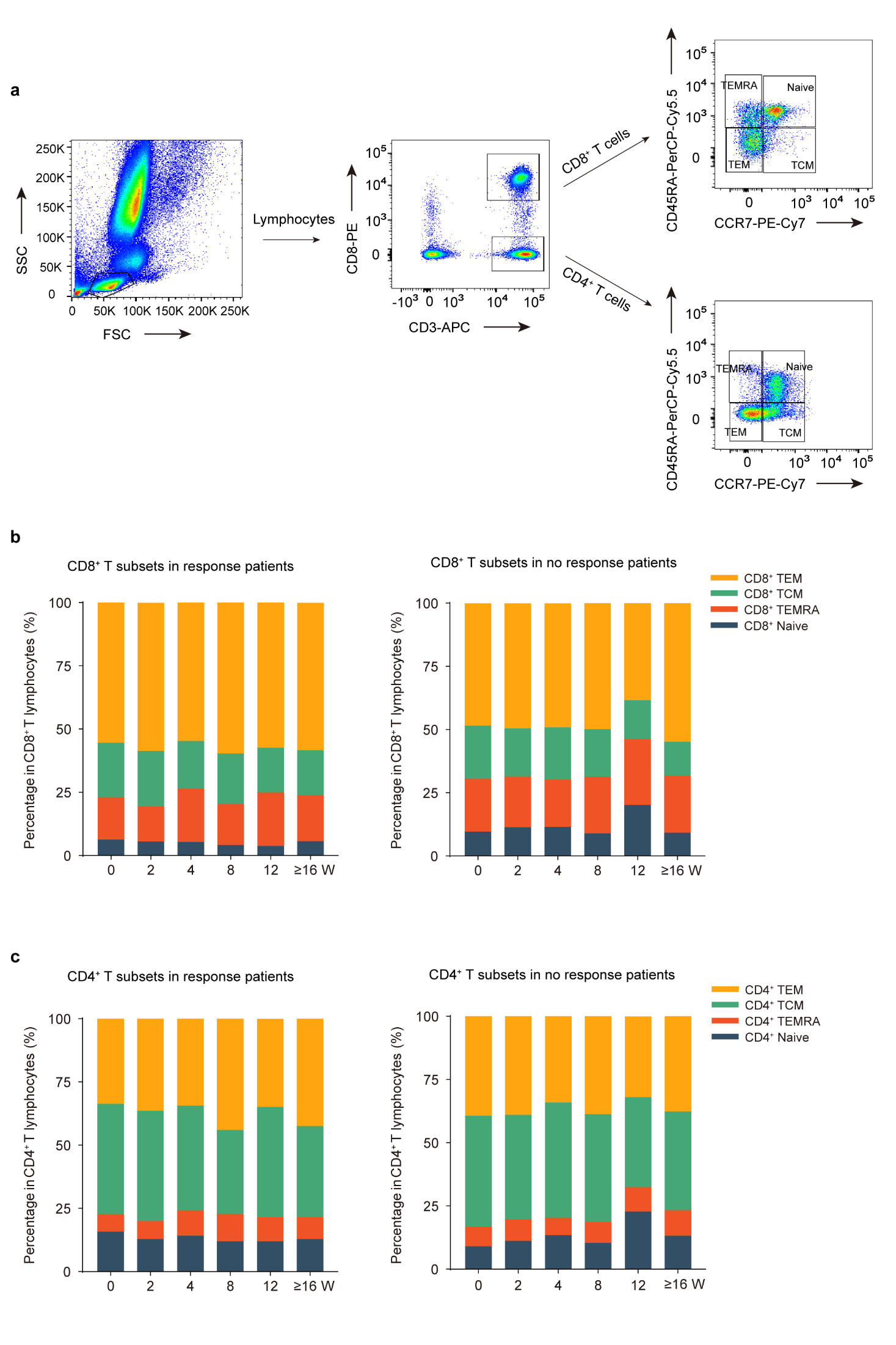
**

**(a)** The flow gating strategy of T cell subsets: according to the expression of CCR7 and CD45RA, CD4^+^ T cells and CD8^+^ T cells were divided into TEMRA (CCR7^-^CD45RA^+^), Naïve (CCR7^+^CD45RA^+^), TCM (CCR7^+^CD45RA^-^) and TEM (CCR7^-^CD45RA^-^) four subgroups. **(b)** After UC-MSC infusion, the percentage of CD8^+^ naïve T cells and TCM cells had decreased in response group, this change was not found in the no response group. **(c)** Dynamic changes in CD4^+^ T subsets were not obvious in both two group after UC-MSCs infusion. Data are mean± s.e.m in stacked column chart (n=8 in response group, n=8 in no response group). Data are mean ± s.e.m (n=8 in response group, n=8 in no response group). W: weeks.

**Fig. S8: Dynamic changes in the proportion of B cell subset and suppressor B cells in the response and no response group after UC-MSC infusion.**

**
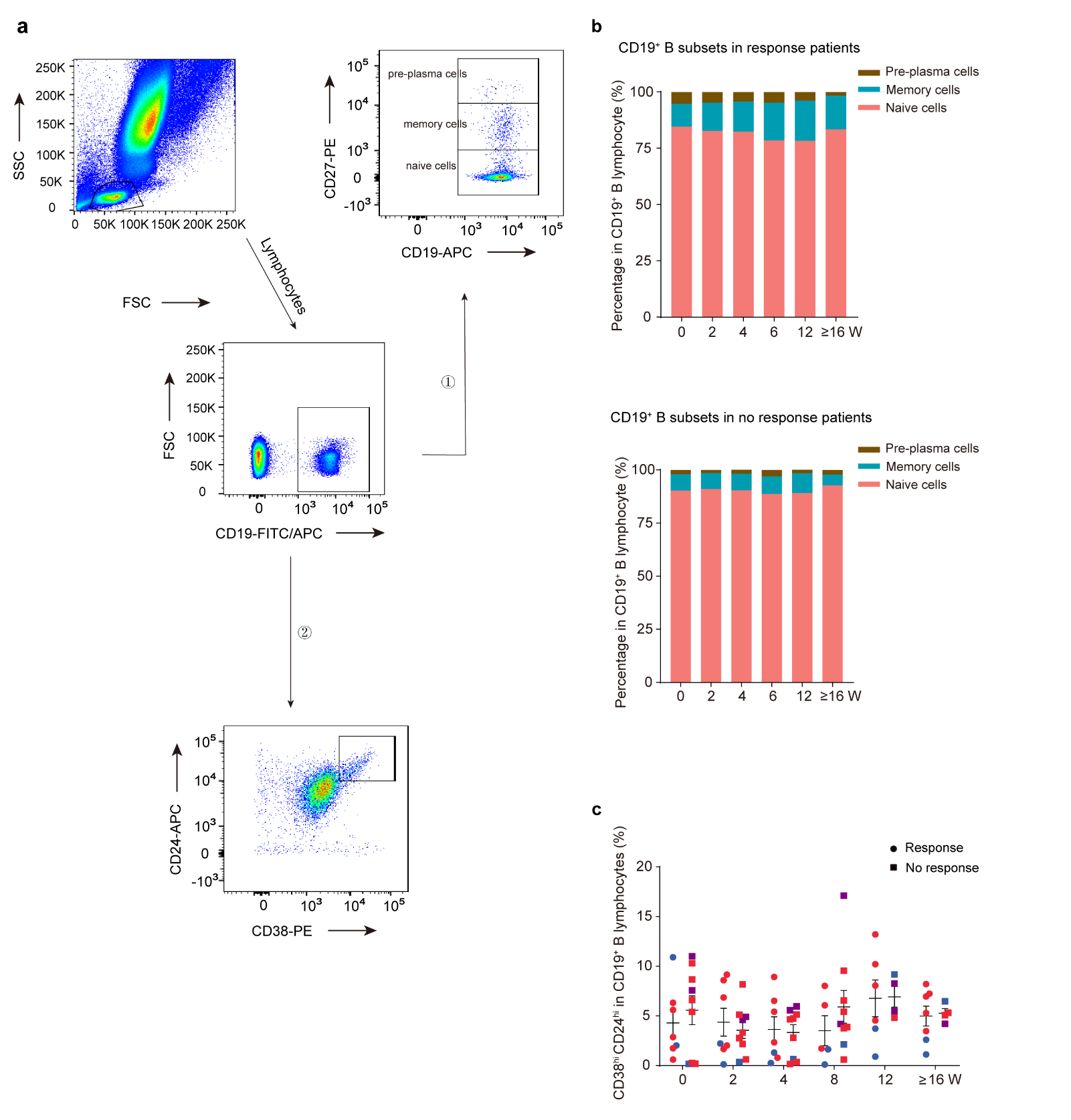
**

**(a)** The flow gating strategy of B cell subsets and suppressor B cells. CD19^+^ B cells were divided into three subpopulations according to the expression intensity of CD27 on the surface: naïve cells (CD27^-^), memory cells (CD27^dim^), and pre-plasma cells (CD27^high^). Define the cells which highly expressed CD24 and CD38 as the suppressive B cells. **(b)** After UC-MSCs infusion, the percentage of CD19^+^ memory cells had slowly increased, and the proportion of naïve B cells decreased in the response group (upper). Changes of B cell subsets were not significant in the no response group (lower). **(c)** The percentage of CD38^hi^CD24^hi^ cells in CD19^+^ B cells had a slightly decrease in the response and no response group. Data are mean± s.e.m in stacked column chart (n=8 in response group, n=8 in no response group). Data are mean in scatterplot (n=7 in response group, n=9 in no response group), different colours represent different infusion doses of UC-MSCs in the scatterplots: purple=0.5×10^6^ cells/kg, green=1.0×10^6^ cells/kg, red=2.0×10^6^ cells/kg. W: weeks.

1. **Supplemental tables**

**Table S1. Demographic characteristics of patients in 3 dose cohort**

| **Characteristics** | | **Total patients (n=18)** | **Dose escalation phase**  **(n=12)** | | | **Dose expansion phase**  **(n=6)** |
| --- | --- | --- | --- | --- | --- | --- |
|  |  |  | **0.5×10^6^ cells/kg (n=3)** | **1.0×10^6^ cells/kg (n=3)** | **2.0×10^6^ cells/kg (n=6)** | **2.0×10^6^ cells/kg**  **(n=6)** |
| **Age (y), median (range)** | | 44 (30-56) | 34 (30-53) | 44 (40-54) | 48 (39-53) | 43 (39-56) |
| **Sex, n (%)** | | | | | | |
|  | Female | 15 (83.3) | 1 (33.3) | 3 (100.0) | 6 (100.0) | 5 (83.3) |
|  | Male | 3 (16.7) | 2 (66.7) | 0 (0.0) | 0 (0.0) | 1 (16.7) |
| **Duration of thrombocytopenia (months), median (range)** | | 73 (15-360) | 84 (15-360) | 120 (24-192) | 74 (15-108) | 40.5 (27-172) |
| **Baseline platelet count (×10^9^/L), median (range)** | | 6 (2-24) | 5 (4-22) | 6 (2-6) | 14 (3-24) | 7 (2-19) |
|  | <10×10^9^/L, n (%) | 10 (55.6) | 2 (66.7) | 3 (100.0) | 2 (33.3) | 3 (50.0) |
|  | 10-30×10^9^/L, n (%) | 8 (44.4) | 1 (33.3) | 0 (0.0) | 4 (66.7) | 3 (50.0) |
| **Previous therapies, n (%)** | | | | | | |
|  | Glucocorticoids | 18 (100.0) | 3 (100.0) | 3 (100.0) | 6 (100.0) | 6 (100.0) |
|  | IVIG | 17 (94.4) | 3 (100.0) | 3 (100.0) | 5 (83.3) | 6 (100.0) |
|  | rhTPO / TPO receptor agonists | 17 (94.4) | 2 (66.7) | 3 (100.0) | 6 (100.0) | 6 (100.0) |
|  | Cyclosporine | 7 (38.9) | 1 (33.3) | 1 (33.3) | 2 (33.3) | 3 (50.0) |
|  | Danazol | 10 (55.6) | 1 (33.3) | 2 (66.7) | 3 (50.0) | 4 (66.7) |
|  | Rituximab | 13 (72.2) | 1 (33.3) | 2 (66.7) | 5 (83.3) | 5 (83.3) |
|  | Splenectomy | 10 (55.6) | 3 (100.0) | 2 (66.7) | 2 (33.3) | 3 (50.0) |
|  | Vincristine | 5 (27.8) | 1 (33.3) | 1 (33.3) | 1 (16.7) | 2 (33.3) |
|  | Decitabine | 3 (16.7) | 1 (33.3) | 1 (33.3) | 1 (16.7) | 0 (0.0) |
|  | Cyclophosphamide | 1 (5.6) | 0 (0.0) | 0 (0.0) | 0 (0.0) | 1 (16.7) |
| **Concomitant medications, n (%)** | | | | | | |
|  | Glucocorticoids | 1 (5.6) | 0 (0.0) | 1 (33.3) | 0 (0.0) | 0 (0.0) |
|  | TPO receptor agonists | 7 (38.9) | 0 (0.0) | 0 (0.0) | 3 (50.0) | 4 (66.7) |
|  | Danazol | 1 (5.6) | 1 (33.3) | 0 (0.0) | 0 (0.0) | 0 (0.0) |
| **Bleeding (WHO bleeding scale, grade 1-4), n (%)** | | 18 (100.0) | 3 (100.0) | 3 (100.0) | 6 (100.0) | 6 (100.0) |
| **Platelet glycoprotein (GP) autoantibodies, n (%)** | | | | | | |
|  | Anti-GP Ⅰb/Ⅸ positive only | 2 (11.1) | 1 (33.3) | 0 (0.0) | 1 (16.7) | 0 (0.0) |
|  | Anti-GP Ⅱb/Ⅲa positive only | 6 (33.3) | 0 (0.0) | 1 (33.3) | 1 (16.7) | 4 (66.7) |
|  | Anti-GP Ⅱb/Ⅲa , GP Ⅰa/Ⅱa double positive | 3 (16.7) | 0 (0.0) | 1 (33.3) | 2 (33.3) | 0 (0.0) |
|  | Negative | 7 (38.9) | 2 (66.7) | 1 (33.3) | 2 (33.3) | 2 (33.3) |

Abbreviations: IVIG, intravenous immunoglobulin; rhTPO: recombination human thrombopoietin

**Table S2. Treatment history of patients in 3 dose cohort**

| **Patient ID** | **Sex** | **Age (y)** | **ITP duration (months)** | **Splenectomy (months)** | **Splenectomy outcome** | **RTX (months from last infusion)** | **RTX efficacy** | **Kinds of previous therapies**# | **List of previous therapies** |
| --- | --- | --- | --- | --- | --- | --- | --- | --- | --- |
| 001 | M | 34 | 360 | 168 | CR, relapse | - | - | 4 | Glucocorticoids, IVIG, CsA, splenectomy |
| 002 | F | 53 | 84 | 48 | CR, relapse | - | - | 5 | Glucocorticoids, IVIG, hetrombopag, danazol, splenectomy |
| 003 | M | 30 | 15 | 10 | CR, relapse | 16 | Failure | 7 | Glucocorticoids, IVIG, RTX, eltrombopag, vincristine, decitabine, splenectomy |
| 004 | F | 54 | 24 | 15 | Failure | 6 | Failure | 9 | Glucocorticoids, IVIG, RTX, rhTPO, eltrombopag, vincristine, decitabine, CsA, danazol, splenectomy |
| 005 | F | 40 | 120 | 30 | Response*, relapse | - | - | 5 | Glucocorticoids, IVIG, eltrombopag, danazol, splenectomy |
| 006 | F | 44 | 192 | - | - | 45 | Failure | 4 | Glucocorticoids, IVIG, RTX, romiplostim, eltrombopag |
| 007 | F | 39 | 21 | 8 | CR, relapse | 14 | Failure | 5 | Glucocorticoids, IVIG, RTX, eltrombopag, splenectomy |
| 008 | F | 51 | 62 | - | - | 12 | Failure | 5 | Glucocorticoids, IVIG, RTX, rhTPO, eltrombopag, danazol |
| 009 | F | 51 | 105 | - | - | 100 | Failure | 6 | Glucocorticoids, IVIG, RTX, rhTPO, eltrombopag, avatrombopag, vincristine, danazol |
| 010 | F | 42 | 15 | - | - | 8 | Failure | 5 | Glucocorticoids, IVIG, RTX, rhTPO, eltrombopag, CsA |
| 011 | F | 53 | 108 | 20 | Response*, relapse | - | - | 5 | Glucocorticoids, rhTPO, decitabine, CsA, splenectomy |
| 012 | F | 45 | 86 | - | - | 78 | Failure | 5 | Glucocorticoids, IVIG, RTX, rhTPO, eltrombopag, danazol |
| 013 | F | 42 | 27 | - | - | 18 | Failure | 6 | Glucocorticoids, IVIG, RTX, rhTPO, eltrombopag, avatrombopag, CsA, danazol |
| 014 | F | 44 | 172 | 132 | CR, relapse | 18 | Failure | 8 | Glucocorticoids, IVIG, RTX, rhTPO, eltrombopag, vincristine, CTX, CsA, splenectomy |
| 015 | M | 56 | 49 | - | - | 15 | Failure | 4 | Glucocorticoids, IVIG, RTX, eltrombopag |
| 016 | F | 39 | 32 | - | - | 13 | Failure | 5 | Glucocorticoids, IVIG, RTX, rhTPO, eltrombopag, danazol |
| 017 | F | 55 | 31 | 20 | Failure | 12 | Failure | 8 | Glucocorticoids, IVIG, RTX, rhTPO, eltrombopag, vincristine, CsA, danazol, splenectomy |
| 018 | F | 41 | 127 | 39 | Response*, relapse | - | - | 5 | Glucocorticoids, IVIG, eltrombopag, danazol, splenectomy |

*The maximum platelet counts of patients 005 and 018 after splenectomy were 50×10^9^/L, and the maximum platelet count was 70×10^9^/L in patient 011 after splenectomy.

#The rhTPO and TPO receptor agonists (eltrombopag, hetrombopag, avatrombopag, and romiplostim) were considered the same kind of therapeutic drugs.

Abbreviations: F, female; M, male; IVIG, intravenous immunoglobulin; rhTPO: recombination human thrombopoietin; RTX, rituximab; CsA, cyclosporine; CTX, cyclophosphamide

**Table S3. Treatment outcomes of UC-MSC infusion in 3 dosage cohorts**

| **Outcomes** |  | **Total (n=18)** |  | **Dose escalation phase (n=12)** | | |  | **Dose expansion phase (n=6)** |
| --- | --- | --- | --- | --- | --- | --- | --- | --- |
|  |  |  |  | **0.5×10^6^ cells/kg (n=3)** | **1.0×10^6^ cells/kg (n=3)** | **2.0×10^6^ cells/kg (n=6)** |  | **2.0×10^6^ cells/kg**  **(n=6)** |
| Response, n (%) |  | 8 (44.4) |  | 0 (0.0) | 2 (66.7) | 3 (50.0) |  | 3 (50.0) |
| PLT ≥30×10^9^/L at least doubling of the baseline count at least once, n (%) |  | 9 (50.0) |  | 1 (33.3) | 2 (66.7) | 3 (50.0) |  | 3 (50.0) |
| PLT ≥50×10^9^/L at least once, n (%) |  | 8 (44.4) |  | 0 (0.0) | 2 (66.7) | 3 (50.0) |  | 3 (50.0) |
| PLT ≥100×10^9^/L at least once, n (%) |  | 7 (38.9) |  | 0 (0.0) | 2 (66.7) | 2 (33.3) |  | 3 (50.0) |
| Time of PLT up to 50×10^9^/L, days, median (range) |  | 21 (7-42) |  | - | 21 (21-21) | 14 (7-42) |  | 21 (14-21) |
| Cumulative weeks of PLT ≥30×10^9^/L at least doubling of the baseline count, weeks, median (range) |  | 6.5 (3-27) |  | - | 4.5 (3-6) | 27 (7-27) |  | 6 (3-8) |

Abbreviations: PLT, platelet count

**Table S4. WHO bleeding score before and after UC-MSC treatment**

| **Bleeding Score** |  | **Dose escalation phase (n=12)** | | | | | | | | | | |  | **Dose expansion phase (n=6)** | | | | | |  |
| --- | --- | --- | --- | --- | --- | --- | --- | --- | --- | --- | --- | --- | --- | --- | --- | --- | --- | --- | --- | --- |
|  |  | **0.5×10^6^ cells/kg (n=3)** | | |  | **1.0×10^6^ cells/kg (n=3)** | | |  | **2.0×10^6^ cells/kg (n=6)** | | |  |  | **2.0×10^6^ cells/kg (n=6)** | | |  | | |
|  |  | At enrollment,  n (%) |  | At the end of the 4^th^ week, n (%) |  | At enrollment,  n (%) |  | At the end of the 4^th^ week, n (%) |  | At enrollment,  n (%) |  | At the end of the 4^th^ week, n (%) |  | At enrollment,  n (%) | |  | At the end of the 4^th^ week, n (%) | |  |  |
| 0 |  | 0 (0.0) |  | 2 (66.7) |  | 0 (0.0) |  | 2 (66.7) |  | 0 (0.0) |  | 5 (83.3) |  | 0 (0.0) | |  | 4 (66.7) | | |  |
| 1 |  | 3 (100.0) |  | 1 (33.3) |  | 2 (66.7) |  | 1 (33.3) |  | 6 (100.0) |  | 1 (16.7) |  | 5 (83.3) | |  | 2 (33.3) | | |  |
| 2 |  | 0 (0.0) |  | 0 (0.0) |  | 1 (33.3) |  | 0 (0.0) |  | 0 (0.0) |  | 0 (0.0) |  | 1 (16.7) | |  | 0 (0.0) | | |  |
| 3 |  | 0 (0.0) |  | 0 (0.0) |  | 0 (0.0) |  | 0 (0.0) |  | 0 (0.0) |  | 0 (0.0) |  | 0 (0.0) | |  | 0 (0.0) | | |  |
| 4 |  | 0 (0.0) |  | 0 (0.0) |  | 0 (0.0) |  | 0 (0.0) |  | 0 (0.0) |  | 0 (0.0) |  | 0 (0.0) | |  | 0 (0.0) | | |  |

**Table S5. Rescue therapy in the study**

| **Outcomes** | |  | **Dose escalation phase (n=12)** | | |  | **Dose expansion phase (n=6)** |
| --- | --- | --- | --- | --- | --- | --- | --- |
|  |  |  | **0.5×10^6^ cells/kg (n=3)** | **1.0×10^6^ cells/kg (n=3)** | **2.0×10^6^ cells/kg (n=6)** |  | **2.0×10^6^ cells/kg**  **(n=6)** |
| **Number of patients required, n (%)** | |  | 0 (0.0) | 3 (100.0) | 3 (50.0) |  | 5 (83.3) |
| **Option of rescue therapy** | | | | | | | |
|  | Glucocorticoids |  | 0 (0.0) | 0 (0.0) | 3 (50.0) |  | 0 (0.0) |
|  | IVIG |  | 0 (0.0) | 1 (33.3) | 3 (50.0) |  | 2 (33.3) |
|  | TPO receptor agonists |  | 0 (0.0) | 3 (100.0) | 1 (16.7) |  | 3 (50.0) * |
|  | Platelet infusion |  | 0 (0.0) | 1 (33.3) | 1 (16.7) |  | 1 (16.7) |

*One patient had no response in the previous treatment of eltrombopag (75 mg/d), and continued to administrate for 1.5 years (50 mg/d) until participated in the study. After the MSCs treatment, the patient achieved short-term complete response and relapsed quickly. When increased the dose of eltrombopag to 75 mg/d, the patient achieved CR again. The other two patients were treated with eltrombopag (75 mg/d) as rescue therapy.

**Table S6. Concomitant medications in the study**

| **Patient ID** | **Concomitant medications at baseline** |  | **Concomitant medications after the UC-MSC infusion** |
| --- | --- | --- | --- |
| 002 | Danazol 100mg/d and 200 mg/d alternately for 9 months |  | No change throughout the study |
| 004 | Triamcinolone for more than 1 month with the dosage of 24 mg/d |  | After platelet count increased to ≥100×10^9^/L due to UC-MSCs infusion, triamcinolone tapered to discontinuation;  At the time of relapsed, add eltrombopag 50 mg/d for 1 month, then up to 75 mg/d for 4 months |
| 009 | Avatrombopag 40 mg/d for 6 weeks |  | No change until withdrawal from the study |
| 010 | Eltrombopag 75 mg/d for over 3 months |  | After platelet count increased to ≥100×10^9^/L due to UC-MSCs infusion, the dose of eltrombopag gradually reduced to 25 mg/d |
| 012 | Eltrombopag 75 mg/d for over 3 months |  | No change until withdrawal from the study |
| 015 | No response in the previous treatment of eltrombopag (75 mg/d) for over 3 months, had received eltrombopag (50 mg/d) for 1.5 years at enrollment |  | After MSCs treatment, the patient achieved CR and relapsed. When increased the dose of eltrombopag to 75 mg/d, the patient achieved CR again. |
| 016 | Eltrombopag 75 mg/d for over 2 months |  | No change until withdrawal from the study |
| 017 | Eltrombopag 75 mg/d for over 4 months |  | No change until withdrawal from the study |
| 018 | Eltrombopag 75 mg/d for over 2 months |  | After platelet count increased to ≥100×10^9^/L due to UC-MSCs infusion, eltrombopag tapered to discontinuation |

**Table S7. Pharmacokinetic parameters following a single intravenous administration of UC-MSCs**

| **Patient ID** | **Time points (Cycle threshold value^¶^)** | | | | | | | | | | |
| --- | --- | --- | --- | --- | --- | --- | --- | --- | --- | --- | --- |
|  | -0h～1h | 30min | 1h | 2h | 4h | 8h | 16h | 24h | 48h | 72h | 96h |
| 004 | ND | 37.675 | 39.617 | 37.744 | 37.201 | ND | ND | ND | ND | ND | ND |
| 005 | ND | 38.151 | 38.011 | 37.350 | 38.143 | 38.762 | ND | ND | ND | ND | ND |
| 006 | ND | 38.582 | ND | ND | ND | ND | ND | ND | ND | ND | ND |
| 007 | ND | 36.825 | 36.336 | 37.936 | ND | ND | ND | ND | ND | ND | ND |
| 008 | ND | 36.542 | 36.875 | 37.585 | 38.583 | ND | ND | ND | ND | ND | ND |
| 009 | ND | 38.735 | 38.855 | 38.727 | 38.714 | ND | ND | ND | ND | ND | ND |
| 010 | ND | 37.060 | 38.161 | 38.675 | 38.983 | ND | ND | ND | ND | ND | ND |
| 011 | ND | 37.497 | 39.738 | ND | ND | ND | ND | ND | ND | ND | ND |

Note: ND= none detected.

**^¶^**The numbers in the table represent the Ct values quantified by PCR for the SRY gene. Ct stands for Cycle Threshold, indicating the cycle number at which the fluorescence signal of the amplification product reaches the set fluorescence threshold. Simply put, the Ct value represents the cycle number at which the initial template amplification reaches a certain level of product.

**Table S8. Flow cytometry analysis for ADA before and after UC-MSC infusion**

| **Patient ID** | **Time points (Results)** | |
| --- | --- | --- |
|  | -1h～0h | 48h after the 4^th^ UC-MSCs infusion |
| 004 | Negative | Negative |
| 005 | Negative | Negative |
| 006 | Negative | Negative |
| 007 | Negative | Negative |
| 008 | Negative | Negative |
| 009 | Negative | Negative |
| 010 | Negative | Negative |
| 011 | Negative | Negative |
| 012 | Negative | Negative |
| 013 | Negative | Negative |
| 014 | Negative | Negative |
| 015 | Negative | Negative |
| 016 | Negative | Negative |

Note: ADA= anti-drug antibodies.
