# Supplementary material for: Efficacy and safety of human umbilical cord-derived mesenchymal stem cells in the treatment of refractory immune thrombocytopenia: a prospective, single arm, phase I trial
Source: Signal Transduct Target Ther. 2024 Apr 23;9:102. doi: 10.1038/s41392-024-01793-5 (PMC11039759; doi:10.1038/s41392-024-01793-5)
Supplement: Supplementary file 2 — protocol [file 41392_2024_1793_MOESM2_ESM.docx]

**Institute of Hematology & Blood Diseases Hospital, Chinese Academy of Medical Sciences & Peking Union Medical College**

**Tianjin AmCellGene Engineering Co., Ltd.**

**2021.6.30**

**A Clinical Trial of Umbilical Cord-derived Mesenchymal Stem Cells for the Treatment of Refractory Immune Thrombocytopenia**

**Clinical Trial Protocol**

**Version 3.0**

**Research Protocol Synopsis**

| **Name of cell product** | Umbilical cord-derived mesenchymal stem cells |
| --- | --- |
| **Trial title** | A clinical trial of umbilical cord-derived mesenchymal stem cells for the treatment of refractory immune thrombocytopenia |
| **Trial Objectives** | Primary objective: To observe the incidence of adverse events in patients after umbilical cord-derived mesenchymal stem cell (UC-MSC) infusion, and to preliminarily evaluate the safety of clinical infusion of UC-MSCs.  Secondary objectives: To preliminarily evaluate the effectiveness of UC-MSCs in the clinical treatment of refractory ITP patients through this clinical study. |
| **Trial design** | A single-centre, open-label, single-arm study |
| **Main inclusion criteria** | 1) 18-60 years of age, regardless of gender;  2) Confirmed diagnosis of ITP for at least 6 months with an insufficient response to first-line treatment drugs (IVIG, corticosteroids), thrombopoietin drugs and rituximab in second-line, or failed to response or relapsed after splenectomy;  3) Subjects who received splenectomy or rituximab must have finished the therapy more than 3 months prior to enrollment;  4) Platelet counts < 30×10^9^/L with bleeding;  5) Expected survival time for at least 6 months;  6) The function of liver and kidney is less than 1.5 times the upper limit of normal value, and the physical examination is qualified;  7) ECOG physical status score ≤ 2;  8) Cardiac function: grade 2 or below according to the New York Society for Cardiac function;  9) Voluntarily sign the informed consent. |
| **Timepoint and**  **index of**  **efficacy evaluation** | Detect and record the platelet counts at 1, 2, 3, 4, 5, 6, 7, 8, 9, 10, 11, 12, 16, 20, and 24 weeks after 4 times infusion, evaluate the changes in peripheral blood platelet counts. |
| **Safety evaluation index** | Vital signs of the subjects and adverse reactions related to UC-MSCs infusion such as fever, embolism, vomiting, diarrhea, rash, and hepatitis B, hepatitis C, and other virus infections are observed immediately after the first infusion to the 4th month (16w) visit period. |

、

| **Number of subjects** | At least 15 cases |
| --- | --- |
| **Cell product specification** | Umbilical cord-derived mesenchymal stem cells, mainly composed of human umbilical cord-derived mesenchymal stem cells in vitro, 5mL/bag, containing 2.0×10^7^ cells. The cell cryopreservation solution is a compound electrolyte solution containing 5% human serum albumin and 10% dimethyl sulfoxide (DMSO). |
| **Trial design and protocol** | Following the "3+3" dose escalation design principle, three escalating dose groups A, B, and C were established and 3 subjects were enrolled in each dose group sequentially. Groups A, B, and C received 0.5×10^6^ cells/kg, 1.0×10^6^ cells/kg and 2.0×10^6^ cells/kg respectively, intravenously infused once a week for four weeks (4 times). During the dose-escalation period, the occurrence of adverse reactions in the three groups was observed and dose-limiting toxicity (DLT) was judged. Once dose-limiting toxicity occurs, the investigator will decide to add 3 subjects to the current dose group (i) or the lower dose group (i-1, i≠ the lowest dose group) according to the number of DLT cases to continue observation or stop the trial. According to the safety data of the dose-escalation period, the researchers selected an appropriate dose expansion group (A or B or C) to enroll 6 subjects for the dose-expansion period to further observe the efficacy of UC-MSCs. |
| **operation process of cell product infusion** | Subjects should be given 5mg dexamethasone to prevent post-infusion reactions prior to infusion, and the infusion duration should generally not exceed 1 hour. During infusion, patients' vital signs and adverse reactions should be closely observed. If adverse symptoms occur, infusion speed should be slowed down or stopped as appropriate. |
| **Treatment course** | 28 days |
| **Trial schedule** | - From January 2019 to June 2019, carry out research on project data and complete the launch of the project, including further standardizing the trial process, printing CRFs, clinical diary, informed consent, and other documents, improving the design of the clinical trial protocol, obtaining ethical approval, and preparing for the launch meeting and the work arrangement before clinical trial enrollment; - From June 2019 to June 2021, conduct subject recruitment and clinical study; It is planned to collect 15 enrolled subjects within 24 months; - From June 2021 to December 2021, subjects are followed up, biological samples are tested, and data are collected. - From January 2022 to December 2022: Complete clinical research report and sort out papers. |
| **Follow-up plan and schedule** | Patients will be followed up for 6 months after infusion of umbilical cord-derived mesenchymal stem cells. |

# 1. Title

A clinical trial of umbilical cord-derived mesenchymal stem cells (UC-MSCs) for the treatment of refractory immune thrombocytopenia (ITP)

# 2. Background and Basis

Immune thrombocytopenia (ITP) is an organ-specific autoimmune disease characterized by decreased platelet count and mucocutaneous bleeding due to the increased destruction and impaired production of platelets associated with autoimmunity. Current studies suggest that the immunodeficiency mechanism of ITP is complex and heterogeneous, and the exact pathogenesis is difficult to elucidate. The mechanisms underlying the development of ITP are multi-step processes involving the collaborations of T lymphocytes, B lymphocytes, antigen-presenting cells (APCs), and monocyte-macrophages system. Autoreactive B lymphocytes secrete antiplatelet antibodies, leading to platelet destruction, T lymphocyte activation, and dysplasia of megakaryocytes. In patients with ITP, there is a cytotoxic T cells 1 (Tc1)/ cytotoxic T cells 2 (Tc2) drift and T helper cells 1 (Th1) / T helper cells 2 (Th2) imbalance which caused by Th1 cell hyperpolarization. Regulatory T cells (Tregs) are key cell subsets in maintaining peripheral immune tolerance and can suppress overactivated immune responses. In patients with ITP, not only the proportion of peripheral Tregs is reduced, but also the immunoregulatory function of Tregs is defective. In addition, complement-mediated platelet lysis and cytotoxic T lymphocyte (CTL) -mediated platelet lysis also play important roles in the pathogenesis of ITP.

Mesenchymal stem cells (MSCs) are a group of cells derived from stromal cells, which can be isolated from a variety of tissues, such as bone marrow (BM), adipose tissue, umbilical cord, umbilical cord blood, and synovial tissue. MSCs have various biological properties of highly proliferation in vitro, multilineage differentiation, hematopoiesis supporting, and immune regulation. They are even used as seed cells for tissue engineering and carriers for gene therapy. Research about MSCs has penetrated many medical fields and has become a hot spot in life science research. In addition to the ability of self-renewal and multilineage differentiation, bone marrow-derived mesenchymal stem cells (BM-MSCs) can promote the proliferation and differentiation of hematopoietic stem cells (HSCs) by secreting hematopoietic growth factors or directly contacting with HSCs to maintain the long-term hematopoietic capacity, and it can also exert immunoregulatory effect via different mechanisms to maintain immune homeostasis in the bone marrow environment. Because of the low immunogenicity due to the absence of expressing major histocompatibility complex molecules and co-stimulatory molecules, MSCs can be used for cell therapy without human leukocyte antigen (HLA) matching, and rarely induce allogeneic rejection after infusion. Up to now, MSCs have been widely used in the treatment of autoimmune and inflammatory diseases such as graft versus host disease (GVHD) and systemic lupus erythematosus (SLE). Studies suggest that, MSCs exert immune regulation function mainly through direct cell to cell contact and paracrine behaviors. In vitro experiments have shown that after contacting with T cells, MSCs will secrete a large amount of soluble cytokines (IL-6, TGF-β, IDO) to inhibit the proliferation of T cells, and can also up-regulate the expression of Treg. Besides, MSCs can also regulate the immune balance by interfering with DC maturation or maintaining the proportion of immature DC, inducing Th2 cell differentiation.

Currently, MSCs have been approval for clinical trials in a variety of inflammatory diseases due to their powerful immune regulation function and extremely low immunogenicity. MSCs can effectively reduce the incidence of GVHD after hematopoietic stem cell transplantation, and also have a certain effect on the treatment of aplastic anemia. This provides a theoretical and practical basis for us to use MSCs to improve the immune imbalance in patients with ITP.

Traditional therapies of ITP in adults include first-line treatments (glucocorticoids with or without intravenous immunoglobulin (IVIG)) and second-line treatments (thrombopoietin (TPO), TPO receptor agonists, splenectomy, and immune-suppressive agents (such as Rituximab, Vincristine, and Azathioprine)). As one of the most common hemorrhagic diseases, ITP has a poor response to the current treatment and most patients need long-term medication maintenance treatment to alleviate bleeding symptoms, which seriously affects their quality of life and increases the economic burden on patients. Therefore, for adult ITP patients, especially refractory ITP patients, there is an urgent need for new treatments of solutions to solve this clinical dilemma. At present, some studies at home and abroad have tried to utilize the immunomodulatory function of MSCs to treat ITP patients and achieved certain effects. However, these results are all from small-sample case reports, and lack of credibility. Therefore, we designed this clinical trial to provide a definite clinical basis for the treatment of refractory ITP with UC-MSCs.

# 3. Objective and Endpoints of the Study

## 3.1 Objective of the Study

- Primary objective:

To observe the incidence of adverse events in patients after UC-MSCs infusion, and to preliminarily evaluate the safety of clinical infusion of UC-MSCs.

- Secondary objectives:

1. To preliminarily evaluate the effectiveness of UC-MSCs in the clinical treatment of refractory ITP patients;
2. To preliminarily evaluate the pharmacokinetics characteristic of UC-MSCs in patients with primary ITP;
3. To preliminarily evaluate the immunogenicity of UC-MSCs in patients with primary ITP;
4. To explore the changes in immune function before and after UC-MSC infusion.

## 3.2 Study Endpoints

- Primary endpoint:

Safety endpoint: the safety and tolerance of UC-MSCs in patients with primary ITP. The safety and tolerance of UC-MSCs will be evaluated according to the incidence and severity of adverse events and serious adverse events (according to NCI CTCAE 5.0 standard).

- Secondary endpoints:

Effectiveness endpoints:

1. Platelet counts at each visit point to evaluate the response rate.
2. Percentage of subjects with platelet counts≥50×10^9^/L and ≥100×10^9^/L at least once respectively.
3. Percentage of subjects with platelet counts ≥ 30×10^9^/L and 2-fold increase from baseline at least once.
4. The time required from the first infusion to platelet counts first ≥ 50×10^9^/L.
5. Percentage of subjects receiving emergency treatment
6. Percentage of subjects with reduced concomitant medications.
7. The longest duration of the subject's platelet counts continuously ≥ 30×10^9^/L.
8. Changes in bleeding scores (WHO bleeding scores) throughout treatment.

Pharmacokinetic endpoint:

Pharmacokinetic characteristics of UC-MSCs in patients with primary ITP, especially the concentration changes of UC-MSCs in peripheral blood.

Immunogenicity endpoint:

Incidence of UC-MSCs antibodies.

Other study endpoints:

Changes in immune status in vivo after UC-MSCs infusion.

# 4. Expected Effect

Platelet counts in some subjects show an upward trend and eventually increase significantly after UC-MSC infusion. No obvious adverse events caused by UC-MSC infusion happen, and the vital signs of patients are normal.

# 5. Study Design and Statistical Methods

## 5.1 Study Design

This is a single-centre, open-label, single-arm study, which is divided into two-stage: dose-escalation stage, and dose-expansion stage.

In the dose-escalation stage, three successively increasing dose groups were set up, and the initial dose of the study was the lowest dose group. Subject enrollment, judgment and treatment of dose-limiting toxicity (DLT) refer to the traditional "3+3" dose-escalation design principle. That is, starting from the lowest dose group, 3 subjects were enrolled each time. If no DLT is observed, the trial will be continued at the next level dose; if DLT occurs in one of three patients, three more patients will be enrolled in the current dose, and when no DLT occurs anymore, the trial will be continued at the next level dose. If 2 or more DLTs occur in the 3 or 6 patients, the dose escalation will be stopped, and the current dose will be determined as the maximum tolerated dose (maximum tolerated dose, MTD). The flow of this trial is shown in Figure 1. Considering the safety of UC-MSCs, there will be 9 to 12 patients enrolled in the dose-escalation stage.


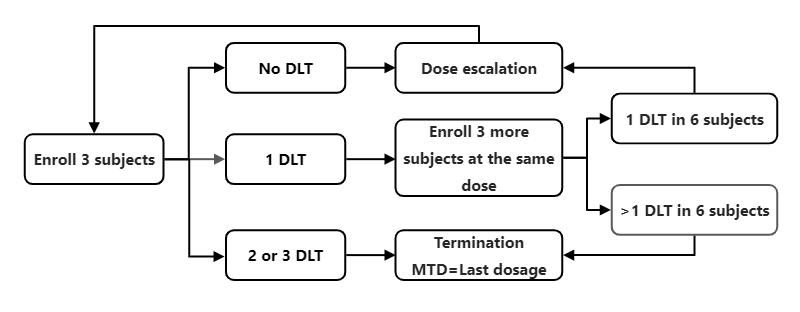
**Figure 1.** “3 + 3” dose-escalation protocol


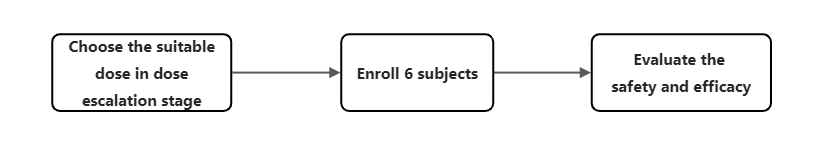
The final dose in the dose-expansion phase will be determined based on the MTD in the dose-escalation phase, safety, efficacy, and opinion considered by the principal investigator, six patients will be enrolled in the dose-expansion stage to preliminarily explore the efficacy.

**Figure 2.** Six subjects will be enrolled to further evaluate the safety and efficacy of this dose infusion of umbilical cord MSCs

**Definitions of Dose Limiting Toxicity (DLT)**

Newly appeared ≥ grade 3 adverse event that is related to UC-MSCs infusion occurred from the start of UC-MSC infusion to 1 month after the last infusion, and cannot be relieved to grade 1 within 72 hours after treatment with drugs or other interventions.

**5.2 Statistical Methods**

### 5.21. Sample Size Estimation

Three dose groups will be set in the dose-escalation stage, 3 subjects will be enrolled each time and 9-12 patients will be enrolled in total according to the "3+3" dose-escalation protocol.

There will be 6 patients enrolled in the dose-expansion stage to explore the efficacy.

### 5.22. Analysis Set

Safety Set (SS): All subjects enrolled who received the trial medication and had at least one subsequent safety visit. The SS will be used for the analysis of safety data.

Full Analysis Set (FAS): All enrolled subjects who received at least one dose of trial medication and had at least one efficacy evaluation. The FAS will be used for the efficacy analysis.

Per Protocol Set (PPS): A subset of the subjects in the FAS who are compliant with major requirements of the clinical study protocol. The PPS will be used for the auxiliary analysis for the efficacy analysis.

### 5.23. Statistical Analysis Methods

- Basic statistical methods

All statistical analyses will be conducted using SPSS 26.0 and GraphPad Prism 9.0 software, statistical analysis will be primarily descriptive. Quantitative data will be presented as median (interquartile range), and qualitative data will be presented as n (%). The differences between groups of quantitative data will be performed using student's t tests or Wilcoxon non-parametric tests. Qualitative variables will be tested with chi-square tests or Fisher's exact tests. The methods of Kaplan-Meier will be used to estimate the time-to-event data. All tests will be calculated with a two-sided significance level of 0.05.

- Safety analysis

Summary tables for adverse events have to include all AEs that occurred in the whole period of study. The correlation between treatment and AEs will be determined by the principal investigator.

- Effectiveness analysis

Proportions with 95% confidence intervals will be calculated for the efficacy endpoint.

# Inclusion, Exclusion Criteria and Assignment Methods

## 6.1 Inclusion Criteria

1. 18-60 years of age;
2. Confirmed diagnosis of ITP for at least 6 months with an insufficient response to first-line treatment drugs (IVIG, glucocorticoids), and thrombopoietic agents (recombinant human thrombopoietin (rhTPO), eltrombopag, avatrombopag, romiplostim, etc.) and anti-CD20 monoclonal antibody (rituximab) in second-line, or failed to splenectomy or subsequent relapse;
3. Subjects who received splenectomy or rituximab must have finished the therapy more than 3 months prior to UC-MSCs inclusion;
4. Platelet counts < 30×10^9^/L with bleeding;
5. Expected survival time for at least 6 months;
6. The function of liver and kidney is less than 1.5 times the upper limit of normal value, and the physical examination is qualified;
7. Subjects are allowed to maintain stable ITP concomitant treatment. The dose of glucocorticoids (less than or equal to 0.5mg/kg prednisone or equivalent dose of glucocorticoids) and thrombopoietin receptor agonists should be stable for at least 4 weeks, and the dose of azathioprine, danazol, cyclosporine A, mycophenolate mofetil, tacrolimus, and retinoic acid should be stable for at least 12 weeks;
8. Be able to understand the purpose and risks of the study and provide informed consent in accordance with national and local privacy regulations;
9. Cardiac function: Grade 2 or below according to the New York Society for Cardiac function;
10. ECOG physical status score ≤ 2.

## 6.2 Exclusion Criteria

1. Subjects with primary disease of important organs (liver, kidney, heart, etc.), or with immune system diseases;
2. Secondary thrombocytopenia caused by various reasons, such as connective tissue disorders, bone marrow hematopoietic failure disease, myelodysplastic syndrome, malignancy, drugs, inherited thrombocytopenia, common variable immune deficiency, lymphoma, etc.
3. Subjects infected with human immunodeficiency virus (HIV);
4. Uncontrollable and active infections during the screening period, including hepatitis B, hepatitis C, cytomegalovirus, EB virus, or positive syphilis antigen;
5. Subjects with extensive and severe bleeding, such as hemoptysis, upper gastrointestinal hemorrhage, intracranial hemorrhage;
6. Subjects with heart disease that requires treatment or hypertension that has been judged by researchers to be poorly controlled currently;
7. Subjects with any venous or arterial thrombosis, atherosclerosis, and other diseases;
8. Subjects with a history of malignant solid tumor or have received allogeneic stem cell transplantation or organ transplantation;
9. Subjects with mental disorders who are unable to sign normal informed consent and conduct trials and follow-up;
10. Subjects whose toxic symptoms caused by pre-trial treatment have not disappeared;
11. Subjects with other serious diseases that may limit their participation in this trial (diabetes; severe cardiac insufficiency; myocardial obstruction or unstable arrhythmia or unstable angina pectoris in the last 6 months; gastric ulcer; active autoimmune disease, etc.);
12. Subjects with septicemia or other irregular bleeding;

## Female subjects who are nursing or pregnant/suspected pregnant (positive pregnancy tests for human chorionic gonadotropin in urine during screening).

## 6.3 Method of Assigning to Groups

Join the group sequentially.

# 7. Number of Subjects

At least 15 patients are expected to be recruited.

Three dose groups will be set in the dose-escalation stage, 3 subjects will be enrolled each time and 9-12 patients will be enrolled in total according to the "3+3" dose-escalation protocol.

There will be 6 patients enrolled in the dose-expansion stage to explore the efficacy.

# 8. Examination items of subjects before and after infusion

1. Basic examination: height, weight, blood pressure, heart rate, etc.
2. Hematological examination: blood routine, blood biochemistry, immune function, etc.
3. Virological examination: hepatitis virus (HBV, HCV), herpes simplex virus, cytomegalovirus (CMV), HIV, Treponema pallidum, etc.
4. 12-lead ECG.
5. Chest X-ray.
6. Ultrasonic examination of abdomen such as liver and spleen.
7. Pharmacokinetic study: A total of 11 blood samples should be collected from each female subject before and 30 minutes, 1h, 2h, 4h, 8h, 16h, 24h, 48h, 72h, 96h after the first infusion of UC-MSCs; all of them are collected from peripheral blood vessels and no less than 2ml. The blood sample collection tubes are anticoagulated with heparin calcium and are taken to mark the subject's medical record number, the time, and the date of blood collection. The collected specimens should immediately be stored in a special location in the refrigerator at 2-8 ℃ and marked.
8. Detection of MSC antibody production: Peripheral blood samples of all subjects are collected before administration and 48 hours after the fourth administration. No less than 2ml of peripheral blood samples are collected each time. The blood sample collection tubes are anticoagulated with heparin calcium and are taken to mark the subject's medical record number, the time, and the date of blood collection. The collected specimens should immediately be stored in a special location in the refrigerator at 2-8 ℃ and marked.
9. Immune function monitoring: The peripheral blood samples of the subjects are collected at the following time points: the day before the UC-MSCs infusion, before the third infusion, 4w, 8w, 12w, 16w, and 24w after the first infusion. Items mainly include the distribution of subsets of Th cells, the proportion of Treg and CD3+CD8+CD28- inhibitory T cells, the expression of costimulatory molecules on the surface of antigen presenting cells, the levels of plasma inflammatory factors and immunoglobulins, as well as the proliferation and activation of T cells and B cells, the killing effect of cytotoxic T lymphocytes on platelets, the induced culture, phenotypic changes, antigen presentation, induction of Treg production of DC cells in vitro.
10. All subjects who received UC-MSCs infusion were followed up for 3-5 years to observe the incidence of long-term adverse events such as tumors.

Check the research flow chart 3 for details.

# Usage, Dosage, Time and Course of Umbilical Cord-derived MSCs

## 9.1 Usage and Dosage of Umbilical Cord-derived MSCs

1. Name of cell products: Umbilical cord-derived MSCs.
2. Specification: 5mL/bag, the main component is MSCs derived from human umbilical cord cultured in vitro, containing 2.0×10^7^ cells. The cell cryopreservation solution is a compound electrolyte solution containing 5% human serum albumin and 10% dimethyl sulfoxide (DMSO).
3. Usage of cell products: Divide into 3 dose-escalation groups (Group A, Group B, and Group C) in turn, with 3 subjects in each group and proceed sequentially from low dose to high dose group according to the principle of dose-escalation. The specific number of subjects in the group is based on a traditional "3+3" dose-escalation protocol, 3 subjects will be enrolled each time, and if no DLT occurs in 3 cases, the trial will be continued at the next level dose; if DLT occurs in 1 of 3 patients, 3 more patients will be enrolled in the current dose, and when no DLT occurs anymore, the trial will be continued at the next level dose. If 2 or more DLTs occur in the 3 or 6 patients, the dose escalation will be stopped, and the current dose will be determined as the MTD.
4. 3 dose-escalation groups- Group A, Group B, and Group C:

- The dose of each infusion in Group A is 0.5×10^6^ cells/kg, intravenous infusion, once a week for a total of 4 times;
- The dose of each infusion in Group B is 1.0×10^6^ cells/kg, intravenous infusion, once a week for a total of 4 times;
- The dose of each infusion in Group C is 2.0×10^6^ cells/kg, intravenous infusion, once a week for a total of 4 times;

According to the above test results, the investigator will determine a specific dose experimental group (one of Group A, B, or C), and expand the sample size of the subjects to 6 cases.

## 9.2 Infusion Process of Umbilical Cord-derived MSCs

Subjects should be given dexamethasone 5mg before the infusion to prevent infusion reactions.

Umbilical cord-derived MSCs are administered by intravenous drip, and the cells need to be resuscitated and diluted 5 times before infusion. The operation method is as follows:

1. Preparation of the required liquid: Aseptically operates in the ultra-clean workbench, extract 20ml/bag of compound electrolyte MG3 injection (Otsuka, 500ml/bag) with a syringe, cover the needle cap, and place them in the ultra-clean bench as a cell diluent for use.
2. Cell recovery: Take the aluminum box containing the cell freezing storage bag out of the refrigerant and quickly place it in a water bath of more than 2 liters at 40 °C (container: 160 mm × 90 mm × 15 mm rectangular box) to quickly melt the cell suspension in the storage bag. After the cell suspension is completely thawed, the cell freezing storage bag is taken out from the water bath, and this process should be completed within 2 minutes. Precautions for this process: Before thawing cells, the water temperature of the water bath shall not be lower than 37°C; during the cell recovery process, it is forbidden to apply mechanical pressure to the unthawed cells to accelerate the thawing process (e.g. pinching by hand); if multiple bags of cells are required, thaw bag by bag; dilution of the cell suspension must be performed immediately after thawing.
3. Cell suspension dilution: After taking out the cell freezing storage bag from the water bath, the cell suspension dilution process is completed aseptically in the ultra-clean workbench. Use dry sterile cotton balls or sterile gauze to wipe off the moisture on the outer surface of the cell freezing storage bag and the mouth of the tube, and then sterilize the tube mouth of the freezing storage bag according to the routine disinfection of infusion. Inject the prepared 20 ml cell diluent slowly through one of the tubes and remove the syringe. Seal the tube mouth with sterile tape, bring the prepared cell bag to the beds as soon as possible, and gently shake the bag to mix the liquid in the bag.
4. Administration: Hang the cell freezing storage bag on the infusion stand, and use a disposable blood transfusion device for intravenous drip in accordance with routine blood transfusion requirements. The initial 10ml infusion rate is 2ml/min, and the remaining volume should be instilled at 4ml/min.
5. Line flushing: Transfer the blood transfusion device to normal saline injection, continue to infuse about 20-50ml intravenously, and completely infuse the remaining cell suspension in the line into the body, and the administration is completed.
6. Notes: all enrolled subjects should use umbilical cord MSCs prepared from the passage cell bank derived from the primary cell of the same donor.

## 9.3 Time

The duration of infusion is generally not more than 1 hour. During the infusion process, the patients' vital signs and adverse reactions should be closely observed. If adverse symptoms occur, the infusion speed should be slowed down or the infusion should be stopped as appropriate.

## 9.4 Treatment Course

Infusions will be administered one time a week, a total of 4 times, and a course is 28 days.

# Management of Infusion Reactions

## 10.1 Delay/Pause/Stop Administration

If subjects experience any of the following adverse events, it is recommended to suspend or discontinue the administration of UC-MSCs to allow the subjects to recover from the toxicity, whether the adverse events are related to the administration or not.

- Criteria for discontinuation of administration are as follows:

1. If severe allergy occurs during the infusion process, such as anaphylactic shock, the administration should be stopped immediately and patients should be withdrawn from the trial;
2. If arterial or venous thrombosis occurs during the infusion process, such as deep vein thrombosis, myocardial infarction, etc., the investigator needs to judge the correlation with the infusion of UC-MSCs, and determines whether to continue the administration;

- Criteria for delay of administration are as follows:

1. Severe bleeding symptoms, such as gastrointestinal hemorrhage, and cerebral hemorrhage;
2. Febrile or infectious neutropenia;
3. Grade 3 or higher non-hematological toxicity, except in the following cases:

- Grade 3 nausea that can resolve with antiemetic treatment within 7 days
- Grade 3 vomiting that can resolve with antiemetic treatment within 7 days
- Grade 3 diarrhea that can resolve with antidiarrheal treatment within 7 days
- Grade 3 fatigue
- Grade 3 Weakness

If any of the above adverse effects occur, it is recommended to pause umbilical cord-derived MSC dosing until recovery to ≤ Grade 2 or baseline; Dosing should be restarted according to treatment schedule (Grade 2 laryngeal edema or Grade 2 bronchospasm requires full recovery).

In addition, if the platelet counts are ≥300×10^9^/L during the infusion, the drug administration can be suspended until the platelet counts are less than 300×10^9^/L, and then the administration can be resumed.

## 10.2 Prevention and Management of Infusion-related Reactions

Subjects should be given dexamethasone 5 mg before infusion of umbilical cord-derived MSCs to prevent infusion reactions.

**Infusion-related reaction*****CTCAE Term**

| **Grade** | **CTCAE Term** |
| --- | --- |
| 1 | Mild transient reaction; infusion interruption not indicated; intervention not indicated |
| 2 | Therapy or infusion interruption indicated but responds promptly to symptomatic treatment (e.g., antihistamines, NSAIDs, narcotics, IV fluids); prophylactic medications indicated for ≤24 hrs |
| 3 | Prolonged (e.g. not rapidly responsive to symptomatic medication and/or brief interruption of infusion); recurrence of symptoms following initial improvement; hospitalization indicated for clinical sequelae |
| 4 | Life-threatening consequences; urgent intervention indicated |
| 5 | Death |

Definition: A disorder characterized by an adverse reaction to the infusion of pharmacological or biological substances.

### 10.2.1 Grade 1 or 2 Infusion-related Reactions

If subjects experience Grade 1 infusion-related reactions, there is no need to interrupt the infusion and add treatment. However, investigators can decide whether to take the same measures as for Grade 2 infusion-related reactions based on the assessment of the actual situation of the patients. If patients have Grade 2 infusion-related reactions:

- - - The infusion should be stopped immediately.
    - Subjects should receive appropriate treatment with H1 receptor antagonists and/or paracetamol or prednisolone (for Grade 1 infusion-related reactions, treatment may be assessed by the investigator on a case-by-case basis).
    - Once symptoms are relieved, the infusion can be continued at an infusion rate not exceeding 50% of the initial rate until complete dosing.

### 10.2.2 Grade 3 Infusion-related Reactions

If subjects experience a Grade 3 infusion-related reactions:

- - - The infusion should be stopped immediately.
    - Subjects should receive appropriate treatment with H1 receptor antagonists and/or paracetamol or prednisolone as clinically needed, with additional drugs added such as epinephrine and bronchodilators if needed.
    - Once symptoms are relieved, the infusion can be continued at an infusion rate not exceeding 50% of the initial rate until complete dosing. If Grade 3 symptoms recur, repeat the above steps. When the Grade 3 infusion-related reactions occur for the third time, subjects should terminate the infusion and should not receive further study drug treatment.

### 10.2.3 Grade 4 Infusion-related Reactions

If subjects experience a Grade 4 infusion-related reaction:

- - - The infusion should be stopped immediately.
    - Subjects should receive appropriate treatment with H1 receptor antagonists and/or paracetamol or prednisolone (as clinically needed), with additional drugs added (e.g., epinephrine, bronchodilators) if needed.
    - Subjects should no longer receive study drug treatment.

# Management of Concomitant Medication or Treatment, Emergency Treatment

## 11.1 Screening Period and Baseline Period (-28d--0d):

Subjects are allowed to maintain stable ITP treatment according to the Chinese ITP treatment guidelines during the screening period and baseline period of the study, including but not limited to glucocorticoids, thrombopoietin receptor agonists, azathioprine, danazol, cyclosporine A and mycophenolate mofetil, tacrolimus, retinoic acid, etc., and the dose of the above drugs should not be increased;

Among them, the dose of glucocorticoids (less than or equal to 0.5mg/kg prednisone or equivalent dose of glucocorticoids) and thrombopoietin receptor agonists should be stable for at least 4 weeks, and the dose of azathioprine, danazol, cyclosporine A, mycophenolate mofetil, tacrolimus, and retinoic acid should be stable for at least 12 weeks;

The start of other drugs for platelet elevation therapy is prohibited, including but not limited to thrombopoietin receptor agonists, rituximab, CD38 monoclonal antibody, and other clinical trial drugs, to avoid a significant impact on efficacy analysis of the umbilical cord-derived MSCs;

## 11.2 Administration Period (0d--4w):

Subjects are allowed to maintain stable ITP treatment according to the Chinese ITP treatment guidelines at the beginning of the study, including but not limited to glucocorticoids, thrombopoietin receptor agonists, azathioprine, danazol, cyclosporine A and mycophenolate mofetil, tacrolimus, retinoic acid, etc., and the dose of the above drugs should not be increased;

Among them, the dose of steroids and thrombopoietin receptor agonists should be stable for at least 4 weeks, and the dose of azathioprine, danazol, cyclosporine A, mycophenolate mofetil, tacrolimus, and retinoic acid should be stable for at least 12 weeks;

The start of other drugs for platelet elevation therapy is prohibited, including but not limited to thrombopoietin receptor agonists, rituximab, CD38 monoclonal antibody, and other clinical trial drugs, to avoid a significant impact on efficacy analysis of the umbilical cord-derived MSCs;

Concomitant ITP medications can be reduced or phased out if subjects have a platelet count ≥50×10^9^/L for at least 2 weeks according to Chinese ITP Guidelines or medical routine.

## 11.3 Follow-up Period (5w-28w):

Concomitant ITP medications can be reduced or phased out if subjects have a platelet count ≥50×10^9^/L for at least 2 weeks after completion of study therapy. Investigators can reduce the concomitant ITP medications according to Chinese ITP Guidelines or medical routine.

After completion of the study treatment, if the subject's platelet count decreases again after a significant increase, the subject's platelet count is less than 30 ×10^9^/L or bleeding symptoms occur, and the investigator judges that the treatment is effective and the case recurs, the investigator could initiate retreatment according to the Chinese ITP Guidelines or medical routine.

## 11.4 Additional Follow-up Period (> 28w):

After the end of the study, patients who have responded to MSC treatment can continue to be followed up to observe changes in platelet levels until relapse. All enrolled patients need to be followed up for a long time (> 2 years) to observe the occurrence of malignant tumors.

## 11.5 Emergency Treatment of ITP

The investigator is permitted to decide whether emergency treatment or rescue therapy for ITP is required at any time with the use of IVIG, platelet transfusion, TPORAs, glucocorticoids, etc.

Any changes in concomitant medication must be recorded in the medical record and case report form (CRF), including the name, dose of the drug, frequency of medication, start and stop dates of medication, and reasons.

# Compassionate use Regimen:

1. Applicable subjects: In this study, subjects who have no obvious adverse reactions after umbilical cord-derived MSCs infusion and respond to the treatment, if the platelet count decreases again, and is judged as relapse by the investigator, and the subjects request another infusion, umbilical cord-derived MSC infusion therapy could be given again;
2. Response (R) is defined as platelet counts ≥ 30 × 10^9^/L after MSCs treatment for at least 2 times (7 days apart), and at least doubling of the baseline count with no bleeding.
3. Relapse is defined as subjects who are judged to respond to treatment after the infusion of UC-MSCs by the investigator, and whose platelet count is found to be less than 30 × 10^9^/L again after follow-up or with bleeding.
4. The inclusion and exclusion criteria, management of infusion and concomitant medications, and emergency treatment for compassionate dosing regimen are consistent with this protocol.
5. Dose of re-infusion: the final dose used in the dose-expansion phase.
6. Infusion time: At least 16 weeks after the first infusion of UC-MSCs in this study.
7. Evaluate the efficacy and safety of umbilical cord-derived MSCs again, complete the pharmacokinetic study, detect the production of MSCs’ antibodies, and monitor the immune function;
8. Continued follow-up time: at least 16 weeks after the first dose of UC-MSC infusion in the supplemental regimen.

# Criteria for Suspension and Termination of Clinical Studies

## 13.1 Investigator-decided Withdrawal

Subject withdrawal from the trial means that the investigator decides to withdraw the case from the trial when the enrolled subject appears unsuitable to continue during the trial.

1. The number of enrolled subjects does not meet the requirements.
2. Subjects have some comorbidities, complications or special physiological changes during the trial and are not suitable to continue the trial.
3. Subjects have poor compliance during the trial.
4. Subjects have adverse events or serious adverse events during the trial and are not suitable to continue the trial.
5. Subjects die.

## 13.2 Subject’s Voluntary Withdrawal

According to the informed consent, the subject has the right to withdraw from the trial. It is also considered withdrawal although the subjects do not explicitly propose to withdraw from the trial, they no longer accept medication and testing and are lost to follow-up. The reasons for their withdrawal such as poor efficacy, intolerant of certain adverse reactions, unable to continue clinical research, economic factors, or failure to follow up without reason should be known as possible and recorded.

## 13.3 Measures of Shedding Cases

The CRF of cases withdrawn for any reason should be kept, the last test results will be considered the final results and will be used for the FAS analysis of their efficacy and adverse reaction. Those who respond to the UC-MSCs before completing the full course of infusion will not be considered as shedding cases but as response ones.

## 13.4 Conditions of Study Suspension

Suspension of the trial means that the clinical trial does not end as planned but stops during the trial. The main purpose of the trial suspension is to protect the rights and interests of the subjects, ensure the quality of the trial, and avoid unnecessary economic losses.

1. If a serious safety problem occurs during the trial, the trial should be suspended in time.
2. The effect of the drug is found too poor or even ineffective and has no clinical value during the trial, the trial should be suspended to avoid delaying the effective treatment of the subjects and at the same time to avoid unnecessary economic losses.
3. The protocol of clinical trial is found to have major mistakes and it is difficult to evaluate the effect of drugs, or a well-designed protocol has a significant deviation in the implementation, and it is difficult to evaluate the effect of drugs if continues.
4. The sponsors request the suspension (such as funding reasons, management reasons, etc.).
5. The administrative departments cancel the test, etc.

A final assessment of the subject should be made at this time and the suspension of study should be recorded on the appropriate page of the CRF. Subjects who withdraw from the trial due to adverse events should be followed up by the investigator.

## 13.5 Conditions of Study Termination

National Medical Products Administration may order the applicant to modify the trial protocol, suspend or terminate the clinical trial under any of the circumstances as follows:

1. The ethics committee fails to perform its duties;
2. Unable to effectively guarantee the safety of the subjects;
3. Fail to report serious adverse events within the prescribed time limit;
4. Evidence to prove that the drugs used in clinical trials are ineffective;
5. Quality problems of the drugs used in clinical trials;
6. Fraud in clinical trials;
7. Other violations of the Quality Management Code for Drug Clinical Trials.

# Evaluation Criteria of Efficacy

## 14.1 Observation period and index

- During infusion:

Evaluate the infusion safety of umbilical cord-derived MSCs. Observe the changes in vital signs during infusion; whether there is fever, nausea, vomiting, etc.; whether there is an allergic reaction; whether there is hemolysis; whether there is shock, and so on. Note that all AE events should be recorded and whether it is related to infusion should be determined by the investigators.

- After transfusion:

Detect platelet counts at 1, 2, 3, 4, 5, 6, 7, 8, 9, 10, 11, 12, 16, 20, and 24 weeks after 4 times infusion, and the changes in peripheral blood platelet count are recorded to evaluate the evaluation of platelet. At the same time, the safety of umbilical cord-derived MSCs is evaluated. Vital signs of the subjects, hepatitis B, hepatitis C, and other virus infections, and adverse reactions related to MSC infusions such as fever, embolism, vomiting, diarrhea, rash, and so on are observed immediately after the first infusion to the 4th month (16w).

- One week to the first month after the first infusion:

Detect the platelet count once a week and record the changes in peripheral blood platelet count. The safety of umbilical cord-derived MSCs is evaluated comprehensively during each infusion. Observe whether there are adverse reactions related to MSC infusion, such as fever, embolism, vomiting, diarrhea, rash, and so on.

## 14.2 Evaluation Criteria of Efficacy

- Main efficacy criteria:

umbilical cord-derived MSCs infusion can increase the platelet count of the subjects, indicating that the drug is effective.

Evaluation criteria of efficacy:

According to the international consensus of ITP:

- complete response (CR): platelet count ≥ 100 × 10^9^ /L and no bleeding after treatment.
- response (R): platelet count ≥ 30 × 10^9^ /L and at least doubling of the baseline count, and no bleeding after treatment.
- No response (NR): platelet count <30 × 10^9^ / L or less than doubling of the baseline count or bleeding after treatment.
- Relapse: platelet count below 30 × 10^9^/L, or less than doubling of the baseline count, or bleeding, after the achievement of CR or R.

When defining a CR or R, it should be tested at least twice with an interval of at least 7 days.

- Safety efficacy criteria:

The subjects have no obvious adverse reactions caused by umbilical cord-derived MSC infusion, normal vital signs, no hepatitis B, hepatitis C, and other virus infections, and no abnormal organ function. There was no significant difference (P > 0.05) in heart, liver, and renal function between objects before and after treatment, indicating that umbilical cord-derived MSCs have no obvious side effects and are safe.

# Record Requirements of Adverse Events & Reports/Measures of Serious Adverse Events

## 15.1 Recording Requirements of Adverse Events

In case of adverse events after infusion, the events of death and serious injury should be reported to the monitoring organization. Serious injury refers to one of the following situations:

- Life-threatening;
- Causing permanent damage to the function or structure of the body;
- Medical intervention must be taken to prevent the outcomes listed above.

The investigator should record the symptoms, signs, and laboratory tests of subjects， as well as the time, duration, degree, medical intervention, and process of damage in the original medical record and CRF, to ensure that the record is true, accurate, complete, timely and legal. The serious adverse event report form should be completed, signed, and dated. The time, reporting method (telephone, fax, or written), and reporting organization should be recorded in the original record.

## 15.2 Reporting Methods and Measures of Serious Adverse Events

1. While observing the efficacy, closely observe the adverse events or unanticipated toxic side effects (including symptoms, signs, and laboratory tests), analyze the causes, make judgments, and follow up with observations and records.
2. For the adverse events that occur during the study period, the symptoms, degree, occurrence time, duration, treatment measures, and process should be recorded in the CRF to evaluate the correlation with the study drug, which should be recorded in detail, signed and dated by the investigator.
3. When an adverse event is found, the observing physician can decide whether to suspend the observation according to the condition. The case of discontinuation due to adverse reaction should be followed up and investigated with detailed records of the treatments and results.
4. In case of serious adverse events in the study, the research unit must immediately take measures to protect the safety of the subjects, and timely report to the drug administration, the sponsor, and the ethics committee. The institution should report to the national and provincial health administration department and Food and Drug Administration.
5. The investigator should follow up to observe and document the outcome of all adverse events and follow up with the subjects who withdraw from the study due to an adverse event until the adverse event is completely resolved. The investigator must determine the relevance of the study drug to the adverse event and provide a basis to support this determination.
6. All clinical examinations or laboratory tests with clinically significant abnormal changes should be filled in the adverse event form and followed up, which needs to be observed at least once a week until normal or back to the baseline level.

# Follow-up Plan and Implementation Method

## 16.1 Follow-up plan

1. Follow-up period

Different subjects should be followed up at different intervals, depending on their condition. Monthly follow-up should be guaranteed for three months after treatment.

1. Follow-up content

Following up should be done by both the Research and Development Institute and the investigators in the hospital in order to assess the treatment efficacy, changes of condition, and recovery after discharge, and to provide professional guidance to the patients when they should return to the hospital for further medical consultation and measures should be taken if their condition changes.

1. Record of follow-up

Completely record the follow-up time, changes in the patient's vital signs, etc.

1. Update the database

After each follow-up, the information should be added to the patient's original treatment information form in time, and the items and contents of the corresponding case records in the database should be updated.

1. Lost to follow-up

The subjects will be considered lost to follow-up if they cannot be contacted for three consecutive visits due to special circumstances such as refusal to visit, transfer of the account, relocation, and no such person.

1. Termination of follow-up

The follow-up can be terminated for subjects who are lost or confirmed dead.

## 16.2 Implementation methods

1. Individualized follow-up visits.
2. Doctor in charge determines which patient requires follow-up, records the time, content, and outcomes of the follow-up in detail, and decides whether to continue the follow-up. In cases of changes in condition, a follow-up is necessary at any time.
3. Standardization, flexibility, and a timely reflection of the subject's condition are required in the follow-up. The follow-up staff must start the follow-up with a full grasp of the latest medical advice and accurate test results. Subjective assumptions, guesses and carelessness are strictly prohibited.
4. The follow-up staff must respect the patient's privacy, be courteous, use standardized language, be impersonal and comply with medical laws.
5. The follow-up physician cannot make a medical judgment only based on the follow-up information. It should be discussed by at least two or more physicians and comprehensively judged by the investigator before making a medical judgment.

# Ethical Requirements

The clinical study will be carried out in accordance with the Declaration of Helsinki (2008 version) and the relevant regulations for the management of clinical study of stem cells in China. The study protocol should be approved by the ethics committee of the institute before the initiation of the clinical study.

The investigators are responsible for providing subjects or their designated representatives with a complete and comprehensive description of the study's objectives, procedures, and possible risks in written form before they are enrolled in this study. Subjects should be made aware of their right to withdraw from the study at any time. The informed consent must be given to each subject prior to enrollment. It is the responsibility of the investigators to ensure that each subject has signed the informed consent before entering the clinical study, which is retained in the study record.

# Quality Control and Quality Assurance

## 18.1 Quality Control

1. Determination of study protocol: The clinical study protocol is discussed and negotiated by all investigators participating in this clinical study, and is submitted to the Ethics Committee for approval after reaching a consensus on revision.
2. Control measures of laboratory quality: The manufacturers of umbilical cord MSCs have standardized testing indicators, standard operating procedures, and quality control procedures.
3. Qualification of investigators: Investigators participating in clinical research must have the professional expertise, qualifications and abilities of clinical research, pass the qualification examination. The study needs relatively fixed personnel.
4. Pre-study training: Through pre-study training, investigators can fully understand the clinical study protocol and the specific contents of each index.
5. The criteria for determining abnormalities in laboratory tests are based on the normal reference range of the test unit.
6. All observations and findings in clinical studies should be verified to ensure the data’s reliability and that the conclusions in clinical studies are derived from the original data. Ensure that there are corresponding data management measures in the clinical study and data processing stages.
7. Proactive measures should be taken for possible dropouts to control the shedding rate within 20%.
8. The investigators should ensure that the data are entered into the CRF in a correct (consistent with the actual situation of the subjects), complete (no missing items), clear (neat handwriting and easy to identify), and timely manner according to the original observation records of the subjects.

## 18.2 Quality Assurance

1. Institute of Hematology & Blood Diseases Hospital & Chinese Academy of Medical Sciences is responsible for the implementation of the whole clinical study and solving related problems.
2. The sponsor appoints monitors to ensure that the rights and interests of the subjects in the clinical study are protected, the data of the study records and reports are accurate, correct and complete, and the study follows the approved protocol, *Management of Stem Cell Clinical Research* and relevant laws and regulations.

# Responsibilities of the Parties and Publication of Papers

The investigator and responsible institute of the study are required to take up the corresponding responsibilities in accordance with *Management of Stem Cell Clinical Research*, *Quality Control and Preclinical Research Guiding Principles of Stem Cell Products*, and this protocol.

Publication requirements: Results of this clinical study should be written and published by our staff (the first author and corresponding author).

Research Flowchart 3

# Attached table: Flow chart of a clinical trial on "Umbilical cord-derived MSCs for the treatment of refractory ITP"

| **Stage** | **Before enrolling** | **First infusion** | **After the first infusion** | | | | | | | | | | | | | | | | | | |
| --- | --- | --- | --- | --- | --- | --- | --- | --- | --- | --- | --- | --- | --- | --- | --- | --- | --- | --- | --- | --- | --- |
| **Time** |  | **D1** | **W1** | **W2** | **W3** | **W4** | **W5** | **W6** | **W7** | **W8** | **W9** | **W10** | **W11** | **W12** | **W13** | **W14** | **W15** | **W16** | **W20** | **W24** | **W28** |
| **Window period** |  |  | **±**  **2D** | **±**  **2D** | **±**  **2D** | **±**  **2D** | **±**  **2D** | **±**  **2D** | **±**  **2D** | **±**  **2D** | **±**  **2D** | **±**  **2D** | **±**  **2D** | **±**  **2D** | **±**  **2D** | **±**  **2D** | **±**  **2D** | **±**  **2D** | **±**  **5D** | **±**  **5D** | **±**  **5D** |
| **Informed consent** | √ |  |  |  |  |  |  |  |  |  |  |  |  |  |  |  |  |  |  |  |  |
| **Determination of inclusion and exclusion criteria** | √ |  |  |  |  |  |  |  |  |  |  |  |  |  |  |  |  |  |  |  |  |
| **Filling in the basic information** | √ |  |  |  |  |  |  |  |  |  |  |  |  |  |  |  |  |  |  |  |  |
| **Concomitant disease** | √ |  |  |  |  |  |  |  |  |  |  |  |  |  |  |  |  |  |  |  |  |
| **Concomitant medication** | √ | √ | √ | √ | √ | √ | √ | √ | √ | √ | √ | √ | √ | √ | √ | √ | √ | √ | √ | √ | √ |
| **Vital signs [1]** | √ | √ | √ | √ | √ | √ |  |  |  |  |  |  |  |  |  |  |  |  |  |  |  |
| **Blood routine test [2]** | √ | √ | √ | √ | √ | √ | √ | √ | √ | √ | √ | √ | √ | √ | √ | √ | √ | √ | √ | √ | √ |
| **Blood chemistry test [3]** | √ |  |  |  |  | √ |  |  |  | √ |  |  |  | √ |  |  |  | √ |  |  |  |
| **Virological examination [4]** | √ |  |  |  |  | √ |  |  |  |  |  |  |  |  |  |  |  | √ |  |  |  |

| **Stage** | **Before enrolling** | **First infusion** | **After the first infusion** | | | | | | | | | | | | | | | | | | |
| --- | --- | --- | --- | --- | --- | --- | --- | --- | --- | --- | --- | --- | --- | --- | --- | --- | --- | --- | --- | --- | --- |
| **Time** |  | **D1** | **W1** | **W2** | **W3** | **W4** | **W5** | **W6** | **W7** | **W8** | **W9** | **W10** | **W11** | **W12** | **W13** | **W14** | **W15** | **W16** | **W20** | **W24** | **W28** |
| **Window period** |  |  | **±**  **2D** | **±**  **2D** | **±**  **2D** | **±**  **2D** | **±**  **2D** | **±**  **2D** | **±**  **2D** | **±**  **2D** | **±**  **2D** | **±**  **2D** | **±**  **2D** | **±**  **2D** | **±**  **2D** | **±**  **2D** | **±**  **2D** | **±**  **2D** | **±**  **5D** | **±**  **5D** | **±**  **5D** |
| **ECG** | **√** |  |  |  |  |  |  |  |  |  |  |  |  |  |  |  |  | **√** |  |  |  |
| **Chest x-ray** | **√** |  |  |  |  |  |  |  |  |  |  |  |  |  |  |  |  | **√** |  |  |  |
| **B-ultrasound [5]** | **√** |  |  |  |  |  |  |  |  |  |  |  |  |  |  |  |  | **√** |  |  |  |
| **Adverse events** |  | **√** | **√** | **√** | **√** | **√** | **√** | **√** | **√** | **√** | **√** | **√** | **√** | **√** | **√** | **√** | **√** | **√** | **√** | **√** | **√** |
| **Infusion time of MSC** |  | **√** | **√** | **√** | **√** |  |  |  |  |  |  |  |  |  |  |  |  |  |  |  |  |
| **Pharmacokinetic study [6]** |  | **√** |  |  |  |  |  |  |  |  |  |  |  |  |  |  |  |  |  |  |  |
| **Detection of MSC antibody [7]** | **√** |  |  |  | **√** |  |  |  |  |  |  |  |  |  |  |  |  |  |  |  |  |
| **Immune function monitoring [8]** | **√** |  |  | **√** |  | **√** |  |  |  | **√** |  |  |  | **√** |  |  |  | **√** |  | **√** |  |

## Vital signs: Body temperature, pulse, respiration, and blood pressure.

## Blood routine test: Hemoglobin, red blood cell count, white blood cell count, absolute value of neutrophils, platelet count.

## Blood biochemistry test: ALT, AST, GGT, TP, ALB, TBIL, DBIL, GLU, CREA, BUN; immune function test according to the subject's condition.

## Virological examination: Hepatitis B, HCV, HIV, syphilis antibodies, EB virus antibodies, cytomegalovirus antibodies, herpes simplex virus antibodies, etc.

## B-ultrasound: B-ultrasound of liver, gallbladder, pancreas, spleen, and kidney.

## Pharmacokinetic study: A total of 11 blood samples should be collected from female subjects before and 30 minutes, 1h, 2h, 4h, 8h, 16h, 24h, 48h, 72h, 96h after the first infusion of umbilical cord MSCs; all of them are collected from peripheral blood vessels and no less than 2ml. The blood sample collection tubes are anticoagulated with heparin calcium and are taken to mark the subject's medical record number, the time, and the date of blood collection. The collected specimens should immediately be stored in a special location in the refrigerator at 2-8 ℃ and marked.

## Detection of MSC antibody production: Peripheral blood samples of all subjects are collected before administration and 48 hours after the fourth administration. No less than 2ml of peripheral blood samples are collected each time. The blood sample collection tubes are anticoagulated with heparin calcium and are taken to mark the subject's medical record number, the time, and the date of blood collection. The collected specimens should immediately be stored in a special location in the refrigerator at 2-8 ℃ and marked.

## Immune function monitoring: The peripheral blood samples of the subjects are collected at the following time points: one day before the umbilical cord MSCs infusion, before the third infusion, 4w, 8w, 12w, 16w, and 24w after the first infusion. Items mainly include the distribution of subsets of Th cells, the proportion of Treg and CD3+CD8+CD28- inhibitory T cells, the expression of costimulatory molecules on the surface of antigen presenting cells, the levels of plasma inflammatory factors and immunoglobulins, as well as the proliferation and activation of T cells and B cells, the killing effect of cytotoxic T lymphocytes on platelets, the induced culture, phenotypic changes, antigen presentation, induction of Treg production of DC cells in vitro.

## The revised version of the protocol:

| The versionof protocol | Date of protocol | The time of passing the Ethical requirements | Correction |
| --- | --- | --- | --- |
| 1.0 | 2019.3.19 | 2019.4.10 | Original plan |
| 2.0 | 2020.12.1 | 2020.12.21 | Change the inclusion criteria as follows: |
|  |  |  | Before the change: Confirmed diagnosis of ITP for at least 6 months with no response or relapsed after splenectomy and insufficient response to conventional therapy like corticosteroids.  After the change: Confirmed diagnosis of ITP for at least 6 months with an insufficient response to first-line treatment drugs (IVIG, corticosteroids), and thrombopoietin drugs and rituximab in second-line, or no response or relapsed after splenectomy. |
|  |  |  | Reasons for change: The ICR consensus in 2019 pointed out that due to the continuous emergence of new drugs, fewer and fewer patients with ITP have been treated by splenectomy, and the concept of refractory ITP has been abolished. The latest GUIDELINES of ITP in China in 2020 redefine the definition of refractory ITP, that is, it refers to patients who are still diagnosed with ITP after diagnostic reevaluation after insufficient response to first-line treatment drugs, thrombopoietin drugs and rituximab in second-line, or no response or relapsed after splenectomy. Therefore, the inclusion criteria for this clinical trial were modified to match the latest guideline definitions in China. |
|  |  |  | Before the change: > 3 months after splenectomy.After the change: >3 months after splenectomy; >3 months after rituximab treatment. |
|  |  |  | Reason for change: To reduce the impact of rituximab treatment on the clinical trial. |
|  |  |  | Before the change: The peripheral blood samples of the subjects are collected at the following time points: the day before the UC-MSC infusion, before the third infusion, 4w, 8w, 12w, 16w, and 28w after the first infusion. After the change: The peripheral blood samples of the subjects are collected at the following time points: the day before the UC-MSC infusion, before the third infusion, 4w, 8w, 12w, 16w, and 24w after the first infusion. |
|  |  |  | Reason for change: Due to the impact of COVID-19, some subjects cannot finish the follow-up successfully. |
| 3.0 | 2021.6.30 | 2021.7.6 | Add compassionate dosing regimen |
|  |  |  | Reason: Some subjects do not have better treatment methods and request re-infusion, which has been approved by the ethics committee. |
